# Accurate prediction of metagenome-assembled genome completeness by MAGISTA, a random forest model built on alignment-free intra-bin statistics

**DOI:** 10.1186/s40793-022-00403-7

**Published:** 2022-03-05

**Authors:** Gleb Goussarov, Jürgen Claesen, Mohamed Mysara, Ilse Cleenwerck, Natalie Leys, Peter Vandamme, Rob Van Houdt

**Affiliations:** 1 Microbiology Unit, Belgian Nuclear Research Centre (SCK CEN), Mol, Belgium; 2 Laboratory of Microbiology and BCCM/LMG Bacteria Collection, Faculty of Sciences, Ghent University, Ghent, Belgium; 3 Department of Epidemiology & Biostatistics, Amsterdam UMC, VU University, Amsterdam, The Netherlands

**Keywords:** Software, Metagenomics, Binning, Quality control, Alignment-free, DNA mock metagenome

## Abstract

**Background:**

Although the total number of microbial taxa on Earth is under debate, it is clear that only a small fraction of these has been cultivated and validly named. Evidently, the inability to culture most bacteria outside of very specific conditions severely limits their characterization and further studies. In the last decade, a major part of the solution to this problem has been the use of metagenome sequencing, whereby the DNA of an entire microbial community is sequenced, followed by the in silico reconstruction of genomes of its novel component species. The large discrepancy between the number of sequenced type strain genomes (around 12,000) and total microbial diversity (10^6^–10^12^ species) directs these efforts to de novo assembly and binning. Unfortunately, these steps are error-prone and as such, the results have to be intensely scrutinized to avoid publishing incomplete and low-quality genomes.

**Results:**

We developed MAGISTA (metagenome-assembled genome intra-bin statistics assessment), a novel approach to assess metagenome-assembled genome quality that tackles some of the often-neglected drawbacks of current reference gene-based methods. MAGISTA is based on alignment-free distance distributions between contig fragments within metagenomic bins, rather than a set of reference genes. For proper training, a highly complex genomic DNA mock community was needed and constructed by pooling genomic DNA of 227 bacterial strains, specifically selected to obtain a wide variety representing the major phylogenetic lineages of cultivable bacteria.

**Conclusions:**

MAGISTA achieved a 20% reduction in root-mean-square error in comparison to the marker gene approach when tested on publicly available mock metagenomes. Furthermore, our highly complex genomic DNA mock community is a very valuable tool for benchmarking (new) metagenome analysis methods.

**Supplementary Information:**

The online version contains supplementary material available at 10.1186/s40793-022-00403-7.

## Background

In recent years, the importance of metagenome research has come to light, as it has the ability to assess a bacterial gene pool and uncover novel bacterial genomes that cannot be grasped by current laboratory culturing techniques [1, 2] or that originate from poorly understood environments, as shown in the Tara Oceans [3] and Tara Pacific [4] studies on marine environments. Such data is critical to expand our understanding of microbial diversity on Earth, which is estimated to range from 10^6^ [5] to 10^12^ [6] species, of which only a small fraction (approximately 20,000) has been validly named, with roughly 60% having a genome-sequenced type strain [7]. The ability to sequence all microbial genomes within an environmental sample, which is provided by metagenome sequencing, is therefore key to a better understanding of microbiomes. As metagenome sequencing data consists of DNA sequence fragments from multiple species and strains, often numbering in the thousands and from different domains of life, the main challenge in this type of analysis is to properly determine the true origin of each DNA sequence fragment. A reference-based approach, implemented by a variety of tools [8], can be used for well-known environments, such as the human microbiome. However, even if the high computational costs—in both memory and time—associated with alignment are ignored, the quality of the resulting metagenome-assembled genomes (MAGs) heavily depends on the quality of the reference, which includes the accuracy of genome sequence and its annotation in publicly available sequence databases. The alternative to reference-based approaches is de novo reconstruction of MAGs, which typically requires reads to be assembled into contigs, then grouped together into single-taxon bins and further refined. Examples of present tools for each step are SPAdes [9] and Megahit [10] for assembly, MetaBAT [11] and GroopM [12] for binning, and MetaWrap [13] and DAS Tool [14] for bin refinement. More extensive lists of binners and bin refiners can be found in [15, 16]. Since reference-free binning approaches rely on heuristics to group contigs into MAGs, they are prone to error and their results should be carefully scrutinized.

Furthermore, in the absence of a reference, assessing the quality of a MAG is a non-trivial task. Presently, the preferred approach is through detection of known single-copy marker genes (SCMGs). For this purpose, a commonly used tool is CheckM [17], which relies on 43 conserved SCMGs. Other tools, such as BUSCO [18], EvalCon [19] and Anvi’o [20], rely on the same principle, though implementation details—like the exact set of SCMGs and similarity thresholds—differ. However, there are at least two potential issues related to the use of SCMGs. The first is that this approach is limited by its reference when using clade-specific marker genes, which may be of poor quality, too distant or not available for certain MAGs. The second is that it only covers a limited fraction of assembled MAGs, which is particularly small when only relying on universal SCMGs. As a result, SCMGs may be missing in the MAG in a way that is not proportional to the actual fraction of the genome that is absent. Meanwhile, the non-analysed fraction is ignored even though it could provide additional information. One recent tool, GUNC [21], has been developed to address this issue. For this tool, a reference-based approach built on high-quality genomes is used as baseline for estimating the taxonomy of contigs, with a model that estimates contamination parameters built on top of it.

Although GUNC claims to address the shortcoming of CheckM, it is still ultimately a gene-centric approach with an explicit set of reference genomes. To overcome the shortcomings of gene-centric reference-based approaches as well as the overestimation of MAG quality by SCMG-based approaches, we present an alternative de novo-based approach that utilizes information from the whole bin. In order to properly assess our method, as well as illustrate the drawbacks of reference-based tools, we constructed a highly complex DNA mock, consisting of 227 bacterial strains of multiple phyla and with varying levels of similarity. This high complexity serves as a substitute for real metagenomic data, while still providing a ground truth. Although real metagenomes are estimated to contain up to thousands of genomes, which is considerably more than the 227 strains used here, the presented mock is considerably more complex than other gDNA mocks and bypasses potential issues of read sets generated in silico. Indeed, simulation tools are still unable to fully capture the full extent of errors that occur in real sequencing data [22] and efforts to improve them are ongoing, even for well-established technologies such as Illumina [23].

## Methods

### Datasets

The input data for the training datasets was generated by pooling even amounts (by mass) of genomic DNA from 227 bacterial strains (Additional file 1: Table S1), covering the major phylogenetic lineages [24]. Sequencing was performed on the Illumina Novaseq 6000 platform using 2 × 150 bp paired-end sequencing by Baseclear, using their in-house pipeline (Leiden, The Netherlands). Selected bacterial strains were cultured and genomic DNA was extracted as outlined in [25]. Briefly, either a modification of the procedure of Pitcher et al*.* [26], Gevers et al*.* [27] and Wilson [28] or a Maxwell® 16 Tissue DNA Purification Kit were used, after a prior enzymatic lysis step in case of gram-positive strains. DNA integrity and purity were evaluated on a 1.0% (w/v) agarose gel and by spectrophotometric measurements at 234, 260 and 280 nm, respectively. Prior to pooling, DNA concentration was determined with the QuantiFluor® ONE dsDNA System (Promega Corporation, Madison, WI, USA).

The test datasets were constructed from five publicly available short read subsets (Table 1). Four of these consist of reads from genomic DNA mock communities of relatively low complexity [29–32]. The Quince dataset contains simulated reads from complete genomes [33] and is considerably more complex as it contains twice as many genomes as the other test subsets combined. In order to provide a comprehensive overview, we evaluated the performance of CheckM and MAGISTA on the individual test datasets as well as the combined test dataset consisting of all five subsets.

**Table 1:** Datasets used in this study

Dataset	Name^a^	Complexity^b^	Input material^c^	Sequencing output	Read source^d^	Assembly tool^e^	Binning method	Binning parameters^f^
Training	HC227_Cc	227	gDNA evenly	2 × 150 bp PE total: 60 Gb	ERS5705986	SPAdes	CONCOCT	comp
	HC227_Ccc							comp + cov
	HC227_Xcc						MaxBin	comp + cov
	HC227_Mc						MetaBAT2	comp
	HC227_Mcc							comp + cov
Test	BMock12_Mc	12	gDNA unevenly	2 × 150 bp PE total: 64 Gb	SRR8073716	SPAdes	MetaBAT2	comp
	BMock12_Mcc							comp + cov
	Rinke_Mc	54	gDNA evenly	2 × 150 bp PE Total: 13 Gb	Rinke et al. [31]^b^	SPAdes	MetaBAT2	comp
	Rinke_Mcc							comp + cov
	MBARC-26_Mc	26	gDNA unevenly	2 × 150 bp PE total: 51.9 Gb	SRR3656745	SPAdes	MetaBAT2	comp
	MBARC-26_Mcc							comp + cov
	ZymoCS_Mc	10	gDNA evenly	2 × 150 bp PE total: 3 Gb	ERR2984773	SPAdes	MetaBAT2	comp
	ZymoCS_Mcc							comp + cov
	Quince_Mc	210	Simulated reads unevenly	2 × 150 bp PE total: 180 Gb	Quince et al. [33]^b^	MEGAHIT	MetaBAT2	comp
	Quince_Mcc							comp + cov

^a^Letter code after underscore refers to binning method (upper case) and parameters (lower case)

^b^Number of strains in the mock

^c^gDNA: genomic DNA, (un)evenly specifies the distribution of the individual inputs

^d^SRR (Sequence Read Archive accession number), ERR (European Nucleotide Archive accession number)

^e^SPAdes version 3.14, For MEGAHIT, assemblies were provided with the publication

^f^Comp, composition; cov, coverage

The read libraries from all datasets were assembled using SPAdes 3.14 [9] with the –meta flag and subsequently binned using either CONCOCT [34], MaxBin [35] or MetaBAT2 [11]. For CONCOCT and MetaBAT2, binning was based on composition only and on composition and coverage (Table 1). Information on the coverage was generated by realigning the reads to contigs with Bowtie2 [36] and summarized to contigs with the ‘jgi_summarize_bam_contig_depths’ program of MetaBAT2. Binning was performed with and without coverage information because we expected mostly equal coverage for our datasets, and using coverage information may therefore result in over-splitting of genomes into multiple bins (Additional File 2: Table S2).

### Predictor variables

**Fig. 1:**
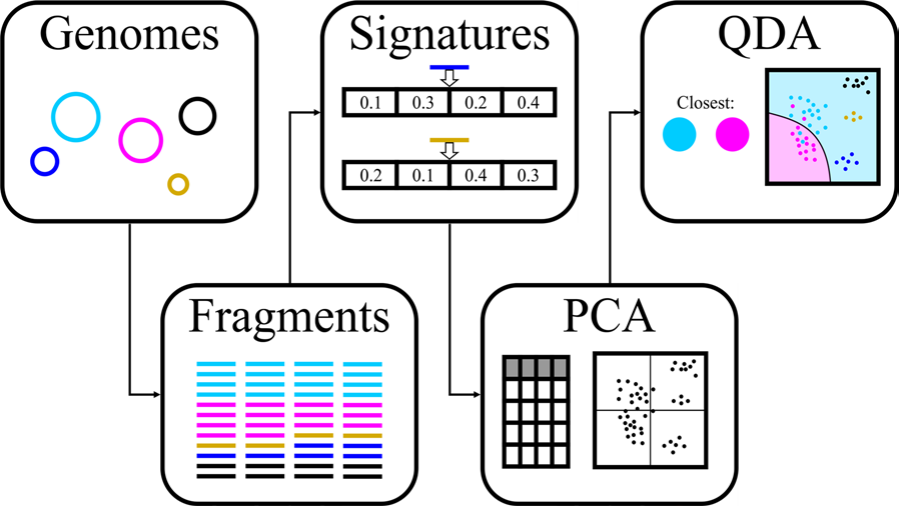
Graphical summary of the pre-processing steps used to evaluate the usability of a specified combination of fragment length and signature choice for a given set of five genomes. Genomes are split into fragments of a specified length and with specified overlap. For each fragment, each signature calculated using the target method is viewed as an observation and PCA is performed to reduce to two dimensions. Finally, QDA is performed between the two closest clusters made up of observations from the same genome and the accuracy of this classifier is produced

In order to assess bin quality, we identified several reference-independent descriptive variables for each bin to be used as predictor variables. To obtain these data, we first split each contig within each bin into fragments of fixed length and then computed all-against-all distances between fragments within a bin using four different methods, i.e. PaSiT4, MMZ3, MMZ4 and Freq4. PaSiT4 is a parameter-dependent method based on tetranucleotide Karlin signatures that was originally optimized for inter-genome distances [25]. Here, it was implemented with a threshold (0.05) optimized for the selected fragment length following the same procedure as described in [25]. The MMZ3 and MMZ4 methods refer to z-scores derived from a second-order Markov model using tri- and tetranucleotides, respectively, and is similar to the approach used in TETRA [37]. Finally, Freq4 refers to normalized correlation coefficients of tetranucleotide frequency profiles. For each method, a specific fragment length was selected in order to produce distinct signature distributions for distinct organisms (see “Results and discussion”, Table 2). We considered fragments with length 1, 5, 10, 20, 30, 40, 50, 75 and 100 kb. The final fragment length for each method was selected through an optimization process that was done on four 5-genome sets from different phyla (Additional file 2: Table S3). Each set was designed such that at least two genomes were from the same family and two genomes were from the same order but from different families. These genomes were artificially split into fragments of the desired length and the target signature was computed for each fragment. For each set of five genomes, all fragments were mixed and principal component analysis (PCA) was performed based on their signatures. For each genome, this procedure generated a distinct distribution along the principal components associated with all five genomes. Quadratic discriminant analysis [38], performed using “qda” from the R [39] package MASS [40], was used to generate a classifier aimed at distinguishing the two genomes with the most overlap within each set. This classifier was limited to using only two principal components. A graphical overview of this approach is presented in Fig. 1. The accuracy of this classifier was used as an indicator of the usability of each combination of fragment length and signature for a given set. These accuracies were averaged across all sets and the resulting value was used to select the final combinations of method and fragment length, taking into account the need to cover both short and long fragments.

**Table 2:** Average accuracy of a quadratic discriminant model between the two most difficult to separate genomes within a set of five genomes

Method	Size (kb)								
	1	5	10	20	30	40	50	75	100
PaSiT4	0.61	0.62	0.65	0.71	0.74	0.79	**0.86**	0.88	0.92
MMZ3	0.65	0.78	**0.84**	0.90	0.92	0.94	0.95	0.97	0.99
MMZ4	0.60	0.76	**0.86**	0.92	0.93	0.95	0.97	0.95	0.96
Freq4	0.71	**0.85**	0.90	0.94	0.95	0.95	0.95	0.96	0.97

Selected combinations are in bold

After the fragment length was selected for each method (Table 2), the distribution of distances using the average, standard deviation, skewness, kurtosis and median, as well as the 2.5, 5, 10, 90, 95 and 97.5% percentiles were calculated. In addition, GC content distributions of 1 kb fragments were also calculated to ensure information availability regardless of contig length. Finally, the bin fraction used and the number of comparisons performed for each method were included, along with the bin size, totalling to 66 predictor variables. These variables constitute the input for the model used to estimate bin quality.

### Choice of model type and input

The 66 predictor variables were log-transformed and PCA was performed on the result for the training set. For a test set, the same log-transformation was applied and the results were then projected onto the principal components derived from the training dataset. The Metagenome-assembled genome intra-bin statistics assessment (MAGISTA) tool is based on a random forest model [41] that predicts bin statistics, such as completeness and purity (see next section), from the original untransformed and projected variables. If some of the variables could not be computed due to the absence of sufficiently long fragments, an alternate model that follows the same principle but does not depend on these missing variables was used instead. This approach enabled us to include all bins containing at least one contig longer than 2 kb or multiple contigs longer than 1 kb in the estimation of bin statistics, which are the minimum required in order to get at least three fragments from which to compute signatures.

The entire procedure, starting from the predictor variables, was implemented in R (v 4.0.3). Random forests were trained using the “RandomForest” function from the package “RandomForest” [42] with default parameters. PCA, including log transform, was performed using an adapted version of “mpm” from the package “mpm” [43]. Linear regression was performed using the “lm” function from the base R library.

### Bin quality assessment (target variables)

The target variables assess the quality of a bin, which is typically done by completeness and purity metrics, from which an F1 score can be computed. These metrics can be computed accurately if the actual reference is known, as is the case with mock metagenomes, i.e. all members are known. We implemented the procedure described in [44] to generate “gold standard” binning results. More concretely, we used MetaQUAST [45] with unique mapping enabled to link each contig to individual references, followed by the AMBER tool [15] that identified the best matching genome for each bin and computed the number of base pairs (bps) associated with that genome. Based on this value, we computed bin completeness, the fraction of a reference genome present in a bin, and bin purity, the fraction of the bin represented by that genome.

$$\text{Bin completeness}=\frac{\text{Matching bps}}{\text{Genome size}}$$

$$\text{Bin purity}=\frac{\text{Matching bps}}{\text{Bin size}}$$

F1 scores were computed using the bin completeness as a measure for recall and the bin purity as a measure of precision:

$${\text{F}}1=2\times\frac{{\text{Recall}}\times {\text{Precision}}}{{\text{Recall}}+{\text{Precision}}}$$

In CheckM, contamination in the validation set was defined as the completeness of the (single) contaminating genome.

$$\text{CheckM contamination }\sim \frac{\text{Mismatched bps}}{\text{Mismatched genome size}}$$

However, because CheckM contamination is based on marker-gene redundancy, it is possible for the predicted value to be (considerably) above 100%. In an effort to make graphs more readable, we derived a “Purity” value for CheckM using the following formula that can be directly converted to and from the CheckM contamination value:

$$\text{CheckM "Purity"}=100\times \frac{100}{100+\text{CheckM contamination}}$$

This conversion provided a good estimation of the actual purity of bins for HC227 in cases where CheckM contamination was greater than 5%.

### Method evaluation

We evaluated the performance of the different methods using two parameters: percentage of explained variance (${R}_{y\sim x}^{2}$) and root-mean-square error (RMSE) with regards to the actual values.

The following formula was used to compute the ${R}_{y\sim x}^{2}$ value:

$${R}_{y\sim x}^{2}=1-\frac{\sum {\left({y}_{i}-{x}_{i}\right)}^{2}}{\sum {\left({x}_{i}-\overline{x}\right)}^{2}},$$

where ${x}_{i}$ is the observed (real) value, ${y}_{i}$ is the value predicted by the model and $\overline{x}$ is the average of all observed values. Note that ${R}_{y\sim x}^{2}$ can become negative when the model-prediction is significantly worse than fixing ${y}_{i}$ to the average value.

## Results

### Generation of a high-complexity genomic DNA mock community

**Fig. 2:**
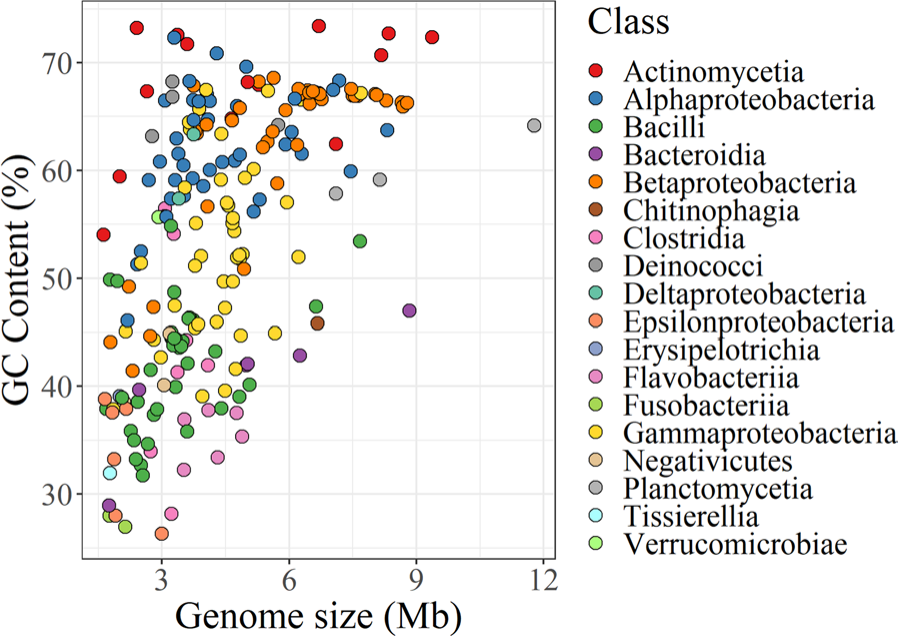
Bacterial strains from the HC227 mock community cover a large range of genome sizes and % GC. Colours indicate the class of each member

In order to define a method for assessing the quality of MAGs, it is necessary to define a training dataset to generate the model. To accomplish this, we created a complex mock community, HC227, consisting of genomic DNA from 227 bacterial strains belonging to 8 phyla (Actinobacteria, Bacteroidetes, Deinococcus-Thermus, Firmicutes, Fusobacteria, Planctomycetes, Proteobacteria and Verrucomicrobia), 18 classes, 47 orders, 85 families, 175 genera and 197 species. The genomes within HC227 cover a large range of sizes (from 1.6 to 11.8 Mb) and % GC (from 26.3 to 73.4%) as well as genome sequence similarity/diversity (Fig. 2; Additional file 3).

**Fig. 3:**
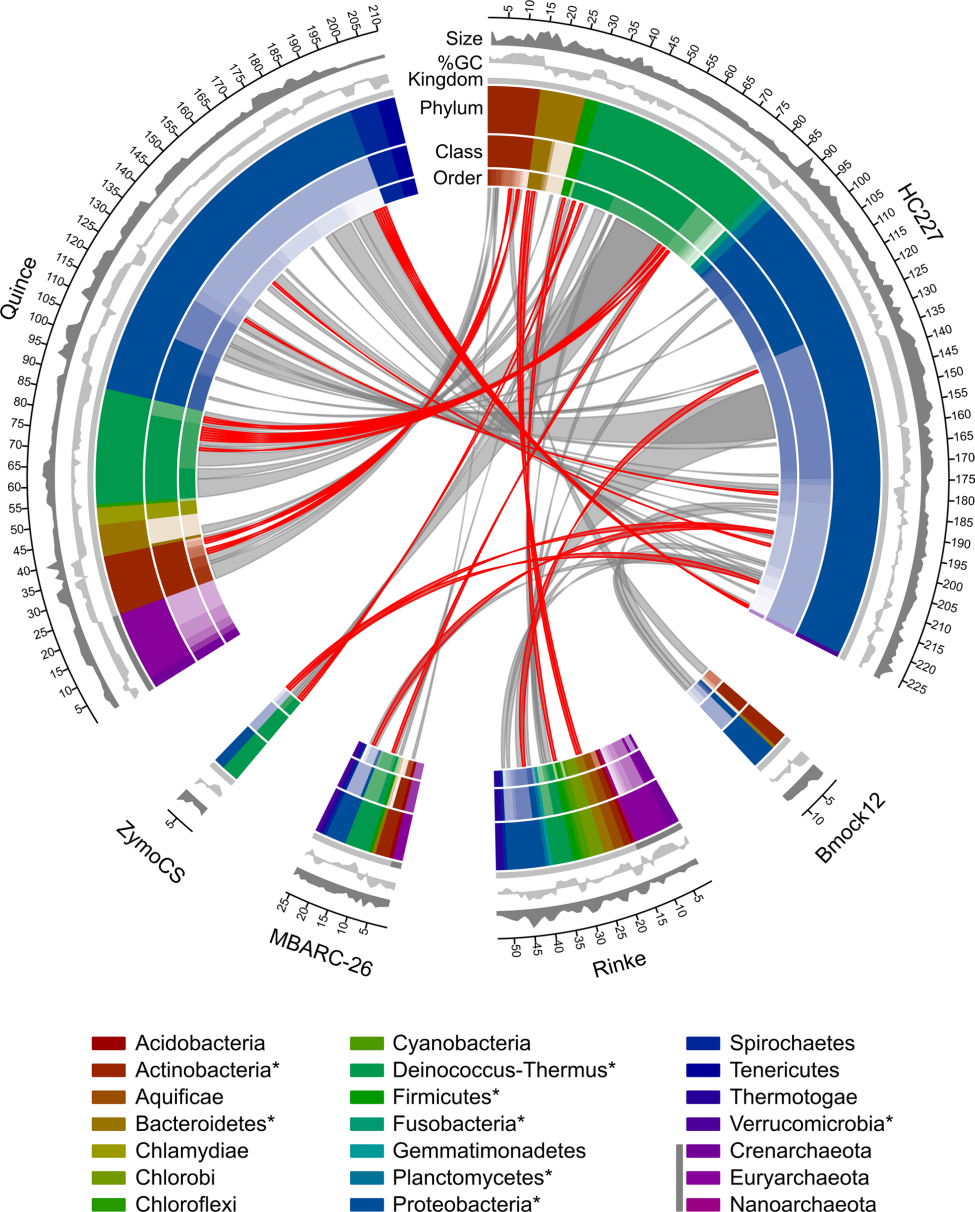
Comparison of the composition of the training (HC227) and test mocks (others). Species (red) and genera (grey) present in HC277 and the test mocks are connected. Each distinct phylum is represented by a separate colour, as are distinct taxonomic classes and orders. Phyla that are present in HC227 are marked with an asterisk in the legend and Phyla belonging to Archaea are indicated by a dark-grey band. Additional information is also provided for each strain in each mock, including its number (outside), genome size (dark grey) and GC content (light grey)

In addition to the HC227 mock, publicly available sequencing data from other well characterised mocks were used for testing. These mocks contained strains that were closely related to those in HC227 as well as strains that belonged to phyla not represented in HC227. A graphical summary of the relations between these mocks and HC227 is presented in Fig. 3.

### Single-copy marker genes for bin quality assessment

**Fig. 4:**
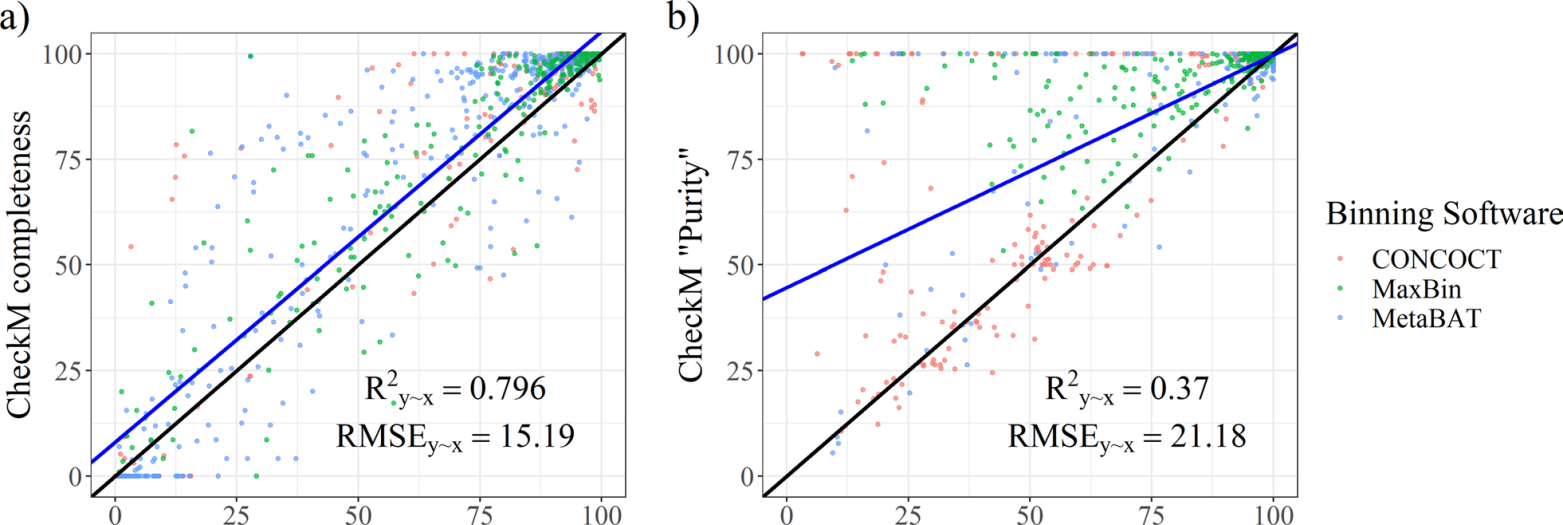
Closest analogues for completeness (**a**) and purity (**b**) obtained from the output of CheckM as a function of the actual values in the training dataset bins. Data points are coloured according to the binner used. The blue line is the best linear fit

Although detection of single-copy marker genes (SCMGs) is the most common strategy to assess bin quality, we found that CheckM (v1.1.2; with its standard 43-SCMG set) overestimated bin quality. In the case of completeness, CheckM prediction generally slightly over-estimated the actual value, and had a relatively high RMSE of 15.19 (Fig. 4a). For purity, which we derived from the predicted contamination using the equation described in “Methods”, this trend was even more pronounced, with many contaminated bins being predicted as near uncontaminated (Fig. 4b). We later also confirmed that these observations were not exclusive to HC227 (see data on test sets below). These observations encouraged us to develop an alternative approach. Recently, another tool called GUNC [21] was developed, claiming to address the inability of SCMGs to predict contamination properly (Additional file 4). However, we found that while it certainly alleviated this problem, this did not lead to an overall improvement in performance and its output also lacks any variable resembling an estimation of completeness (the most relevant variable is probably “reference representation”, shown in Additional file 4).

### Alignment-free intra-bin statistics

We used alignment-free intra-bin statistics to develop a program that predicts the two most commonly used bin metrics, namely completeness and purity.

#### Step 1: optimizing fragment lengths for computing distances

**Fig. 5:**
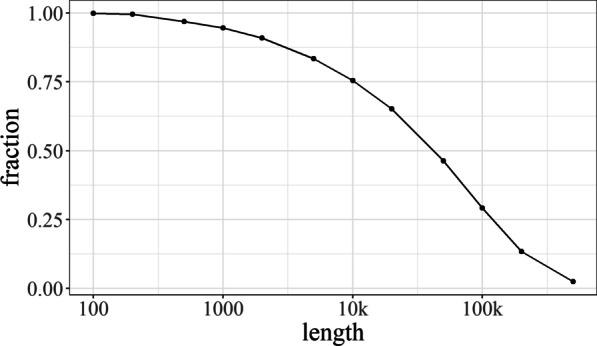
Fraction of the HC227 assembly that can potentially be covered by the analysis for specified fragment lengths

Prior to assessing bin quality using distributions of distances, we first established the optimal fragment sizes for computing these distances. We considered fragments of 1, 5, 10, 20, 30, 40, 50, 75 and 100 kb. These lengths are a trade-off between using a larger fraction of any given bin (shorter fragments) and producing more meaningful inter-fragment distances (larger fragments). The shortest fragments considered were 1-kb fragments, as both CONCOCT and MetaBAT ignore shorter fragments by default. Long fragment length limited the analysis to only a small fraction of the available data. As such, selecting a longer fragment sometimes prevents bin analysis because of the absence of suitably long contigs. The data shown in Fig. 5, which was generated from the training dataset by using the sum of the length of contigs longer than the specified value divided by the total length of all contigs, corroborates the limitation to fragments of at most 100 kb.

However, even with longer fragment lengths, we found that Karlin signatures could not be used to fully separate some species from each other using quadratic discriminant analysis with a fragment length that preserved the majority of the metagenome (Table 2). Finally, for each method we selected a length that offered consistent accuracy, ensured that different methods covered a variety of lengths and did not rely on the presence of excessively long contigs. As a result, sufficiently long contigs produced fragments analysed by all methods, whereas short contigs produced fragments analysed by at least one method.

#### Step 2: calculating bin statistics

**Fig. 6:**
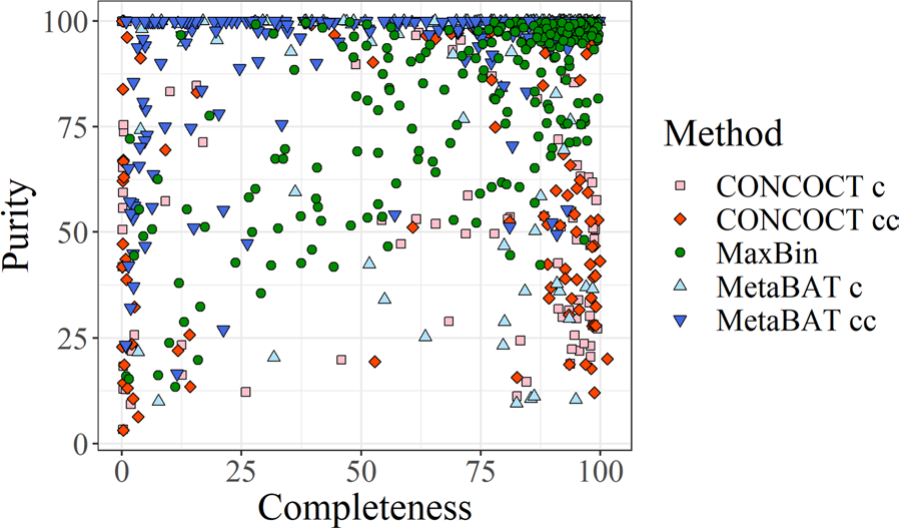
Completeness and purity of bins generated using CONCOCT, MaxBin and MetaBAT2 based on composition (c) and on composition and coverage (cc)

In order to ensure that the final model would be able to deal with any bin, it was necessary to train it using a dataset that covers a wide variety of inputs. As mentioned in the introduction, generating such a dataset by simulation does not represent realistic results accurately, so we used the results of binning software, providing a set of realistic bins of varied quality. For the training dataset, the completeness and purity values of most bins were above 90%. The training dataset was large enough to cover the 2D space formed by all possible combinations of completeness and purity relatively well when combining bins produced by CONCOCT, MetaBAT and MaxBin with different settings (Fig. 6).

#### Step 3: model construction

Distribution parameters for intra-bin distances for all combinations of fragment length and distance computation method selected at the end of Step 1 were used as input to create models for predicting completeness and purity. For some bins, sufficiently long contigs were not available, and the parameters of distributions associated with longer fragment lengths could therefore not be computed. These bins were marked as having incomplete input data (henceforth referred to as “incomplete bins”), which the subsequently created model has to account for. The core of the predictive model relies on random forests, with additional pre-and post-processing steps. For pre-processing, the log-transformed distribution parameters were used to perform principal component analysis and the resulting bin coordinates were added as additional input variables for the random forest. As for the post-processing step, it consisted of a linear regression model derived from a cross-validation analysis of the random forest output for the training dataset. Incomplete bins were removed from the initial training set and the model generated with this data was used whenever a complete bin was provided as part of the testing procedure. For incomplete bins, the same procedure was repeated without the missing predictors. As such, a separate random forest was generated for each of the selected fragment lengths (1, 5, 10 and 50 kb), resulting in four potential models. By using this approach, every bin in the training set could be used and the quality of every bin in the test set could be scored. The training set included bins generated by four binning approaches designed to cover the full range of possible completeness and purity, with 842 bins in total. It contained 675 bins that produced all fragment lengths, 801 bins that produced at least some fragments of 10 kb, and 817 bins that produced some fragments of at least 5 kb. We refer to our procedure as metagenome-assembled genome intra-bin statistics assessment, or MAGISTA. Next to MAGISTA, which relied exclusively on the distance distribution parameters, a model was generated that also included the numeric outputs of CheckM, coined Metagenome-assembled genome intra-bin statistics including CheckM (MAGISTIC). All distribution parameters and alignment-based statistics for each bin in the training and test datasets are provided in Additional file 1 (Tables S4 and S5), along with the relative importance of each input parameter (Additional file 1: Table S6).

### Evaluation of the models

**Fig. 7:**
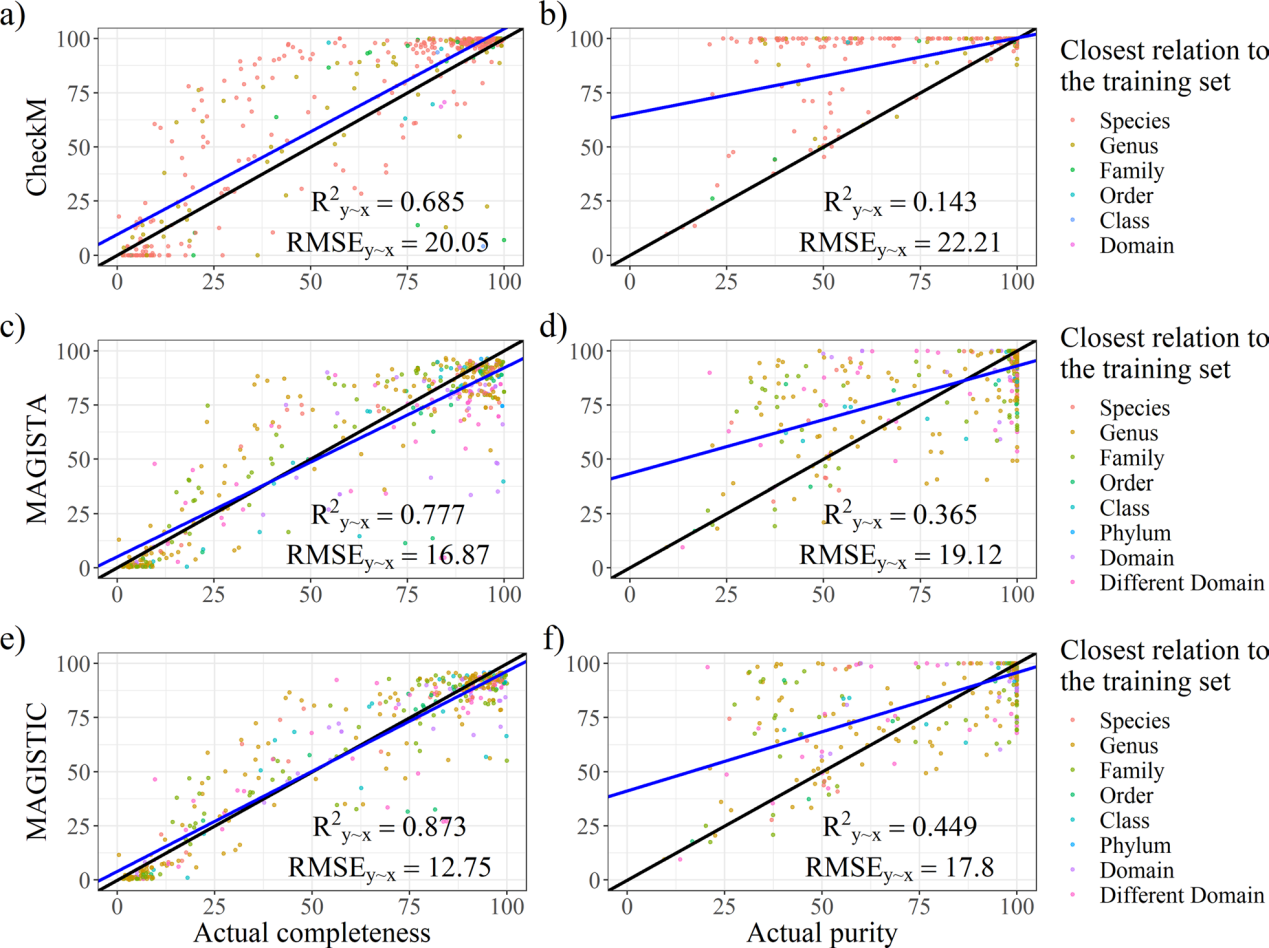
Performance of CheckM (**a** and **b**), MAGISTA (**c** and **d**) and MAGISTIC (**e** and **f**) on all test datasets for completeness (**a**, **c** and **e**) and purity (**b**, **d** and **f**). The black and blue lines indicate the ideal performance and best linear fit, respectively. Data points are coloured in accordance with their taxonomic relation to the most related genome in the training/reference set of the method whose performance is shown

Although we also performed cross-validation (Additional file 5), using publicly available sequencing data from other well-characterised mocks was deemed to be a more representative evaluation test. Here, we report the performance of CheckM, MAGISTA and MAGISTIC on the test datasets. We use the fraction of explained variance (${R}_{y\sim x}^{2}$) and the root-mean-square error (RMSE) (Fig. 7; Table 3) as quantitative measures for performance. For completeness prediction, MAGISTA outperformed CheckM as it achieved a better RMSE (16.87 versus 20.05) and had a higher ${R}_{y\sim x}^{2}$ value (0.777 versus 0.685) (Fig. 7a and c; Table 3). For purity prediction, MAGISTA performed better than the purity value that we derived from CheckM, with an RMSE of 19.12 versus 22.21, and an ${R}_{y\sim x}^{2}$ value of 0.365 vs 0.143 (Fig. 7b and d; Table 3). However, we note that the purity value derived from CheckM is not entirely representative of what CheckM is designed to measure. Nevertheless, it is clear from these results that neither MAGISTA nor CheckM achieved sufficient accuracy to be considered as reliable. MAGISTIC produced better results than MAGISTA (Fig. 7 and Table 3).

**Table 3:** Performance of all models on the test dataset (all) and subsets containing real and simulated reads

Bin statistic	Model	${\mathbf{R}}_{{\mathbf{y}}\sim {\mathbf{x}}}^{{\textbf{2}}}$			RMSE		
		Real	Simulated	All	Real	Simulated	All
Completeness	CheckM	0.744	0.612	0.685	17.28	22.54	20.05
	MAGISTA	0.814	0.730	0.777	14.73	18.81	16.87
	MAGISTIC	0.905	0.836	0.873	10.52	14.68	12.75
Purity	CheckM	0.722	-0.261	0.143	7.74	30.61	22.21
	MAGISTA	0.204	0.240	0.365	13.10	23.76	19.12
	MAGISTIC	0.672	0.234	0.449	8.41	23.85	17.80
F1	CheckM	0.778	0.536	0.666	14.85	23.46	19.58
	MAGISTA	0.787	0.725	0.766	14.57	18.04	16.38
	MAGISTIC	0.884	0.775	0.834	10.75	16.32	13.79

As the test dataset can be subdivided into bins produced from real and simulated reads (Table 1), the performance of the different models was also calculated for these two subsets (Table 3) as well as the individual test datasets (Additional file 2: Table S7). The “real” part consisted of relatively low-complexity metagenomes obtained by mixing DNA of pure cultures, whereas the “simulated” part consisted of the high-complexity simulated metagenome used by Quince et al*.* [33]. The results showed that CheckM performed well for the “real” subset (albeit worse than MAGISTA and MAGISTIC), but poorly for the “simulated” part. MAGISTA and MAGISTIC have a more stable performance. For the sake of completeness, Table 3 also includes F1 scores, which offer a way to compare models using a single value, altough completeness and purity are more relevant to most research questions. Finally, since MAGISTA makes predictions based on the entire genome, it is conceivable that it would be more affected by mobile genetic elements and horizontal gene transfer events than SCMG-based systems. We evaluated this with a case study comparing the presence or absence of broad-host-range plasmids, i.e. RK2 (IncP group), R388 (IncW group) and pIPO2 (PromA group), in the proteobacterial bins of our test dataset. The latter indicated that MAGISTA and, to a lesser extent, MAGISTIC predictions were indeed affected more than CheckM predictions, although the effect was marginal with a 1.05 ± 0.35 and 0.48 ± 0.21 median difference for completeness prediction by MAGISTA and MAGISTIC, respectively (Additional file 2: Table S8).

## Discussion

Metagenomic profiling via assembly and binning, particularly of highly complex samples, relies on performant computational approaches, as was illustrated in the Critical Assessment of Metagenome Interpretation study [44]. However, the optimisation and assessment of these approaches is often done on mock metagenomes of limited complexity or on simulated metagenomes when higher complexity is needed. Ideally, testing should be done with real metagenomes that capture the real bias and nature of the microbiome [46, 47], yet the absolute truth in such samples is unknown. A valid approximation is the use of highly-complex DNA mocks, which are generated by pooling DNA of numerous strains. However, such high-complexity DNA mocks did not exist. Therefore, we started by first generating such a complex mock community that provides a novel and challenging dataset to test metagenomics tools. We elected to use even amounts of DNA for each of the 227 strains. This was done in part to increase the difficulty of binning the mock correctly, thereby simulating the more ambiguous parts of metagenomes with which modern tools still struggle. In addition, it ensured that each genome would have a high likelihood of being completely represented and that the quality of bins would not be linked to their phylogeny.

Assessing the quality of bins is an essential step in data curation and construction of high-quality metagenome assembled genomes. Currently, the most common strategy to assess quality is through the detection of single-copy marker genes (SCMGs), with CheckM being a commonly used tool. The use of SCMGs can yield good results for MAGs derived from known species, where the bin is expected to cover most of the genome. However, when de novo binning is required, for example when analysing poorly studied environments, only 40 [18] to approximately 100 [48] universal SCMGs can be used, which is a fraction of the thousands of genes that bacteria commonly have. In addition, we observed that CheckM (v1.1.2) with its standard 43-SCMG set tended to overestimate both bin purity and completeness, suggesting issues inherent to the method, and motivating the development and implementation of an alternative approach using alignment-free intra-bin statistics.

Figure 4 is a good illustration of the risk associated with relying too heavily on the output of “generic” bin evaluation methods, such as CheckM and GUNC that rely on existing annotated genes, and illustrates the need for complementary methods such as MAGISTA. It also highlights that the construction of high-quality MAGs currently still requires tailored analysis.

MAGISTA is completely independent of existing gene annotation. Instead, distribution parameters for intra-bin distances for all selected combinations of fragment length and distance computation method were used as input to create models for predicting completeness and purity. Random forests, which perform well for noisy data and require very little tuning [38], were selected as the basis of the predictive models. Next to MAGISTA, which relied exclusively on the distance distribution parameters, a model was generated that also included the numeric outputs of CheckM (MAGISTIC).

Both MAGISTA and MAGISTIC outperformed CheckM when it comes to predicting the completeness of bins. Considering the large number of genomes involved in the construction of the CheckM reference, it is difficult to predict how strong its performance degrades when it is exposed to novel taxa, although for the test datasets performance dropped with an increasing number of novel taxa. MAGISTA and MAGISTIC used intra-bin distances and as such uncoupled the genetic makeup of a bin from the parameters used to estimate its quality, thereby creating a method that could perform well regardless of whether the target bin contained known genomes. Nevertheless, we found that the performance of MAGISTA was affected by the relation of the target bin to the training dataset, as bins associated with distant taxa tended to have underestimated completeness (Fig. 7, Additional file 6). To alleviate this issue, the published models could also be trained by including, next to HC227, the test datasets as well as a selection of complete genomes, but we would lack test datasets for estimating the performance of such a model. As for purity, although MAGISTIC is an improvement over CheckM, we do not recommend either MAGISTIC, MAGISTA or CheckM.

Next to dividing the test dataset according to the relatedness of bins to the training set, an intersting case study is to separate the results produced from real and simulated reads. Therefore, the performance of the different models was also calculated for these two subsets (Table 3). The results showed a discrepancy in CheckM performance, with better results  for the real, low-complexity datasets than for the higher-complexity datasets. In contrast, our tools had a more consistent performance. The presence of genomes whose species were not in the reference set for CheckM may also explain the drop in performance for the high-complexity mocks, such as the simulated Quince mock and HC277, as these contain more species that are less closely related to the reference set than the low-complexity mocks. MAGISTA and MAGISTIC have a more stable performance and are thus preferred for more complex cases. The better performance of our methods can be attributed to the complexity, i.e. number and variety of strains, of the DNA mock community we constructed and used in training. This could also indicate that de novo metagenomic analysis tools that are validated using mock communities with a limited number of members are likely to underperform in real situations.

## Conclusion

In this work, we created a novel approach that can be used to predict the quality of metagenome-assembled genomes. This method, MAGISTA, is an equally good alternative to SCMG-based methods for low-complexity metagenomes. For high-complexity metagenomes, it provides a significant improvement over SCMG-based methods, although complexity may not have been the primary factor contributing to this discrepancy. In addition to MAGISTA, we generated an even more accurate prediction with MAGISTIC by incorporating CheckM results. Noteworthy, the error on purity predictions for both SCMG-based and the MAGISTA method is still very high and as such purity predictions should be treated with caution.

Our highly complex genomic DNA mock community accurately captured the complexities and unideal properties of real data, which is not the case for simulated metagenome datasets, and is a very valuable tool for benchmarking (new) metagenome analysis methods, including assembly, binning and taxonomic assignment.

## Supplementary Information

**Additional file 1.** Supplementary Tables S1, S4, S5 and S6. Supplementary Table S1: Bacterial strains in the HC227 mock. Supplementary Table S4: Alignment-based statistics and distribution parameters for the training datasets. Supplementary Table S5: Alignment-based statistics and distribution parameters for the test datasets. Supplementary Table S6: Relative importance of input variables for the Random Forest classifiers used by MAGISTA and MAGISTIC, respectively.

**Additional file 2.** Supplementary Tables S2, S3, S7 and S8 Supplementary Table S2. Number of genome parts counted for the different training datasets; Supplementary Table S3. Bacterial strains used for selecting fragment length for each intra-bin distance calculation method; Supplementary Table S7. Evaluation of bin statistics predicted by CheckM, MAGISTA and MAGISTIC using three different evaluation metrics. Supplementary Table S8. Median absolute difference between predicted values for Proteobacteria bins in the test set, and the same bins to with the addition of the complete sequence of a plasmid.

**Additional file 3.** Graphical comparison of the taxonomy, length, and GC content of all genomes included in the HC227 mock.

**Additional file 4.** Closest analogues for completeness and purity obtained from the output of CheckM (a, b) and GUNC (c, d) as a function of the actual values in the training dataset bins. Data points are coloured according to the binner used. The blue line is the best linear fit.

**Additional file 5.** Cross validation prediction results for HC277.

**Additional file 6.** Variants of Fig. 7 showing the effect of taxonomic distance from the reference or training set on the performance of the model. For each bin, its taxonomic distance from the reference or training set is defined as the taxonomic difference between its best matching genome (see materials and methods) and the closest genome in the reference or training dataset. Each sub-figure corresponds to a “target distance” (i.e. same species, genus, family, order, class or phylum). The six leftmost plots contain only the bins whose distance is exactly the target distance, the six middle plots contain all bins whose taxonomic distance is less than or equal to the target distance, and the six rightmost plots contain only the bins with a taxonomic distance to the reference or training dataset above the target distance.

## Data Availability

Project name: MAGISTA; Project home page: https://github.com/LM-UGent/MAGISTA; Archived version: https://github.com/LM-UGent/MAGISTA/releases/tag/v0.1; Operating system: Linux; Programming languages: bash, R, C; Other requirements: R4.0 or higher, CheckM v1.1.2, GenDisCal v1.1.0.; License: MIT. Sequencing data has been deposited at the European Nucleotide Archive under accession number PRJEB43026. All other data generated or analysed during this study are included in this published article and its additional files.

## References

[1] Steen AD, Crits-Christoph A, Carini P, DeAngelis KM, Fierer N, Lloyd KG, Cameron TJ (2019). High proportions of bacteria and archaea across most biomes remain uncultured. ISME J.

[2] Goh KM, Shahar S, Chan K-G, Chong CS, Amran SI, Sani MH, Zakaria II, Kahar UM (2019). Current status and potential applications of underexplored prokaryotes. Microorganisms.

[3] Bork P, Bowler C, de Vargas C, Gorsky G, Karsenti E, Wincker P (2015). Tara Oceans. Tara Oceans studies plankton at planetary scale. Introduction. Science.

[4] Planes S, Allemand D, Agostini S, Banaigs B, Boissin E, Boss E, Bourdin G, Bowler C, Douville E, Flores JM (2019). The Tara Pacific expedition—a pan-ecosystemic approach of the "-omics" complexity of coral reef holobionts across the Pacific Ocean. PLoS Biol.

[5] Louca S, Mazel F, Doebeli M, Parfrey LW (2019). A census-based estimate of Earth's bacterial and archaeal diversity. PLOS Biol.

[6] Lennon JT, Locey KJ (2020). More support for Earth’s massive microbiome. Biol Direct.

[7] Shi W, Sun Q, Fan G, Hideaki S, Moriya O, Itoh T, Zhou Y, Cai M, Kim S-G, Lee J-S (2021). gcType: a high-quality type strain genome database for microbial phylogenetic and functional research. Nucleic Acids Res.

[8] Breitwieser FP, Lu J, Salzberg SL (2017). A review of methods and databases for metagenomic classification and assembly. Brief Bioinform.

[9] Bankevich A, Nurk S, Antipov D, Gurevich AA, Dvorkin M, Kulikov AS, Lesin VM, Nikolenko SI, Pham S, Prjibelski AD (2012). SPAdes: a new genome assembly algorithm and its applications to single-cell sequencing. J Comput Biol: J Comput Mol Cell Biol.

[10] Li D, Luo R, Liu CM, Leung CM, Ting HF, Sadakane K, Yamashita H, Lam TW (2016). MEGAHIT v1.0: A fast and scalable metagenome assembler driven by advanced methodologies and community practices. Methods.

[11] Kang DD, Li F, Kirton E, Thomas A, Egan R, An H, Wang Z (2019). MetaBAT 2: an adaptive binning algorithm for robust and efficient genome reconstruction from metagenome assemblies. PeerJ.

[12] Imelfort M, Parks D, Woodcroft BJ, Dennis P, Hugenholtz P, Tyson GW (2014). GroopM: an automated tool for the recovery of population genomes from related metagenomes. PeerJ.

[13] Uritskiy GV, DiRuggiero J, Taylor J (2018). MetaWRAP-a flexible pipeline for genome-resolved metagenomic data analysis. Microbiome.

[14] Sieber CMK, Probst AJ, Sharrar A, Thomas BC, Hess M, Tringe SG, Banfield JF (2018). Recovery of genomes from metagenomes via a dereplication, aggregation and scoring strategy. Nat Microbiol.

[15] Meyer F, Hofmann P, Belmann P, Garrido-Oter R, Fritz A, Sczyrba A, McHardy AC (2018). AMBER: Assessment of Metagenome BinnERs. GigaScience.

[16] Yue Y, Huang H, Qi Z, Dou H-M, Liu X-Y, Han T-F, Chen Y, Song X-J, Zhang Y-H, Tu J (2020). Evaluating metagenomics tools for genome binning with real metagenomic datasets and CAMI datasets. BMC Bioinform.

[17] Parks DH, Imelfort M, Skennerton CT, Hugenholtz P, Tyson GW (2015). CheckM: assessing the quality of microbial genomes recovered from isolates, single cells, and metagenomes. Genome Res.

[18] Simão FA, Waterhouse RM, Ioannidis P, Kriventseva EV, Zdobnov EM (2015). BUSCO: assessing genome assembly and annotation completeness with single-copy orthologs. Bioinformatics.

[19] Parrello B, Butler R, Chlenski P, Olson R, Overbeek J, Pusch GD, Vonstein V, Overbeek R (2019). A machine learning-based service for estimating quality of genomes using PATRIC. BMC Bioinform.

[20] Eren AM, Esen OC, Quince C, Vineis JH, Morrison HG, Sogin ML, Delmont TO (2015). Anvi'o: an advanced analysis and visualization platform for 'omics data. PeerJ.

[21] Orakov A, Fullam A, Coelho LP, Khedkar S, Szklarczyk D, Mende DR, Schmidt TSB, Bork P (2021). GUNC: detection of chimerism and contamination in prokaryotic genomes. Genome Biol.

[22] Alosaimi S, Bandiang A, van Biljon N, Awany D, Thami PK, Tchamga MSS, Kiran A, Messaoud O, Hassan RIM, Mugo J (2020). A broad survey of DNA sequence data simulation tools. Brief Funct Genom.

[23] Schmeing S, Robinson MD (2021). ReSeq simulates realistic Illumina high-throughput sequencing data. Genome Biol.

[24] Forterre P (2015). The universal tree of life: an update. Front Microbiol.

[25] Goussarov G, Cleenwerck I, Mysara M, Leys N, Monsieurs P, Tahon G, Carlier A, Vandamme P, Van Houdt R (2020). PaSiT: a novel approach based on short-oligonucleotide frequencies for efficient bacterial identification and typing. Bioinformatics.

[26] Pitcher DG, Saunders NA, Owen RJ (1989). Rapid extraction of bacterial genomic DNA with guanidium thiocyanate. Lett Appl Microbiol.

[27] Gevers D, Huys G, Swings J (2001). Applicability of rep-PCR fingerprinting for identification of Lactobacillus species. FEMS Microbiol Lett.

[28] Wilson K (2001). Preparation of genomic DNA from bacteria. Curr Protoc Mol Biol.

[29] Nicholls SM, Quick JC, Tang S, Loman NJ (2019). Ultra-deep, long-read nanopore sequencing of mock microbial community standards. Gigascience.

[30] Singer E, Andreopoulos B, Bowers RM, Lee J, Deshpande S, Chiniquy J, Ciobanu D, Klenk HP, Zane M, Daum C (2016). Next generation sequencing data of a defined microbial mock community. Sci Data.

[31] Rinke C, Low S, Woodcroft BJ, Raina JB, Skarshewski A, Le XH, Butler MK, Stocker R, Seymour J, Tyson GW, Hugenholtz P (2016). Validation of picogram- and femtogram-input DNA libraries for microscale metagenomics. PeerJ.

[32] Sevim V, Lee J, Egan R, Clum A, Hundley H, Lee J, Everroad RC, Detweiler AM, Bebout BM, Pett-Ridge J (2019). Shotgun metagenome data of a defined mock community using Oxford Nanopore, PacBio and Illumina technologies. Sci Data.

[33] Quince C, Delmont TO, Raguideau S, Alneberg J, Darling AE, Collins G, Eren AM (2017). DESMAN: a new tool for de novo extraction of strains from metagenomes. Genome Biol.

[34] Alneberg J, Bjarnason BS, de Bruijn I, Schirmer M, Quick J, Ijaz UZ, Lahti L, Loman NJ, Andersson AF, Quince C (2014). Binning metagenomic contigs by coverage and composition. Nat Methods.

[35] Wu Y-W, Simmons BA, Singer SW (2016). MaxBin 2.0: an automated binning algorithm to recover genomes from multiple metagenomic datasets. Bioinformatics.

[36] Langmead B, Salzberg SL (2012). Fast gapped-read alignment with Bowtie 2. Nat Methods.

[37] Teeling H, Meyerdierks A, Bauer M, Amann R, Glöckner FO (2004). Application of tetranucleotide frequencies for the assignment of genomic fragments. Environ Microbiol.

[38] Hastie T, Tibshirani R, Friedman J, Hastie T, Tibshirani R, Friedman J (2009). Random forests. The elements of statistical learning.

[39] R Core Team (2020). R: a language and environment for statistical computing.

[40] Venables WN, Ripley BD (2002). Modern applied statistics with S.

[41] Breiman L (2001). Random forests. Mach Learn.

[42] Liaw A, Wiener M (2002). Classification and regression by random forest. R news.

[43] Wouters L, Gohlmann HW, Bijnens L, Kass SU, Molenberghs G, Lewi PJ (2003). Graphical exploration of gene expression data: a comparative study of three multivariate methods. Biometrics.

[44] Sczyrba A, Hofmann P, Belmann P, Koslicki D, Janssen S, Droge J, Gregor I, Majda S, Fiedler J, Dahms E (2017). Critical assessment of metagenome interpretation—a benchmark of metagenomics software. Nat Methods.

[45] Mikheenko A, Saveliev V, Gurevich A (2016). MetaQUAST: evaluation of metagenome assemblies. Bioinformatics.

[46] Motro Y, Moran-Gilad J (2018). Microbial metagenomics mock scenario-based sample simulation (M_3_S_3_). Clin Microbiol Infect.

[47] Fritz A, Hofmann P, Majda S, Dahms E, Dröge J, Fiedler J, Lesker TR, Belmann P, DeMaere MZ, Darling AE (2019). CAMISIM: simulating metagenomes and microbial communities. Microbiome.

[48] Ankenbrand MJ, Keller A (2016). bcgTree: automatized phylogenetic tree building from bacterial core genomes. Genome.

