# Supplementary material for: Accurate prediction of metagenome-assembled genome completeness by MAGISTA, a random forest model built on alignment-free intra-bin statistics
Source: Environ Microbiome. 2022 Mar 5;17:9. doi: 10.1186/s40793-022-00403-7 (PMC8898458; doi:10.1186/s40793-022-00403-7)
Supplement: Supplementary file 2 — Additional file 2. Supplementary Tables S2, S3, S7 and S8 Supplementary Table S2. Number of genome parts counted for the different training datasets; Supplementary Table S3. Bacterial strains used for selecting fragment length for each intra-bin distance calculation method; Supplementary Table S7. Evaluation of bin statistics predicted by CheckM, MAGISTA and MAGISTIC using three different evaluation metrics. Supplementary Table S8. Median absolute difference between predicted values for Proteobacteria bins in the test set, and the same bins to with the addition of the complete sequence of a plasmid. [file 40793_2022_403_MOESM2_ESM.pdf]

Supplementary Table 2. Number of genome parts[a] counted for the different training datasets.

Dataset	Number of genome parts
HC227_Cc	466
HC227_Ccc	518
HC227_Mc	304
HC227_Mcc	366

[a]For each bin, the best matching genome was identified using AMBER. Next, for each bin, all the genomes for which the number of nucleotides was at least 5% of the number of nucleotides assigned to the best matching genome were counted. In this way, when a genome constitutes a non-negligible fraction of a bin, it produces an additional "genome part". In an ideal situation, the number of genome parts should be equal to the number of genomes within the data (here: 227).

Supplementary Table 3. Bacterial strains used for selecting fragment length for each method.

Set	Phylum	Strain	Reads Accession
1	Actinobacteria	*Aeriscardovia aeriphila* LMG 21773	ERS3417869
		Bifidobacterium angulatum LMG 11039	ERS3417870
		Curtobacterium luteum LMG 8787	ERS3417875
		Micrococcus luteus LMG 4050	ERS3417876
		Clavibacter michiganensis LMG 2891	ERS3417874
2	Bacteroidetes	*Bacteroides ureolyticus* LMG 6451	ERS3417886
		Prevotella bivia LMG 6452	ERS3417887
		Marinilabilia salmonicolor LMG 1346	ERS3417888
		Chryseobacterium joostei LMG 18212	ERS3417890
		Epilithonimonas lactis LMG 24401	ERS3417891
3	Firmicutes	*Staphylococcus lentus* LMG 21025	ERS3417913
		Laceyella sacchari LMG 21673	ERS3417914
		Carnobacterium inhibens LMG 23655	ERS3417915
		Enterococcus ureasiticus LMG 26304	ERS3417916
		Tetragenococcus osmophilus LMG 26041	ERS3417917
4	Proteobacteria	*Ensifer saheli* LMG 7837	ERS3417967
		Rhizobium rubi LMG 17935	ERS3417968
		Ancylobacter pratisalsi LMG 29367	ERS3417969
		Maricaulis parjimensis LMG 19863	ERS3417970
		Oceanicella actignis LMG 25334	ERS3417971

Supplementary Table 7. Evaluation of bin statistics predicted by CheckM, MAGISTA and MAGISTIC using three different evaluation metrics.

Evalution Metric[a]	Subset Name	Completeness			Purity			F1 Score		
		CheckM	MAGISTA	MAGISTIC	CheckM	MAGISTA	MAGISTIC	CheckM	MAGISTA	MAGISTIC
R_{lm}^2	Bmock12	0.527	0.884	0.853	0.055	0.804	0.749	0.610	0.874	0.870
	MBARK26	0.934	0.896	0.961	0.702	0.683	0.834	0.917	0.883	0.941
	Rinke	0.829	0.735	0.873	0.936	0.258	0.695	0.865	0.769	0.887
	ZymoCS	0.971	0.938	0.963	0.997	0.700	0.918	0.955	0.871	0.772
	Quince	0.740	0.762	0.841	0.251	0.318	0.373	0.693	0.740	0.795
	Real	0.797	0.816	0.908	0.706	0.516	0.780	0.823	0.831	0.899
	All	0.762	0.795	0.874	0.332	0.373	0.480	0.741	0.786	0.841
$R_{x\sim y}^2$	Bmock12	0.333	0.847	0.842	-0.071	0.290	0.692	0.401	0.848	0.839
	MBARK26	0.918	0.893	0.960	0.689	0.597	0.793	0.898	0.870	0.934
	Rinke	0.725	0.634	0.863	0.934	-0.848	0.469	0.814	0.640	0.864
	ZymoCS	0.964	0.834	0.865	0.996	0.519	0.847	0.940	0.454	0.436
	Quince	0.612	0.755	0.836	-0.261	0.231	0.234	0.536	0.725	0.775
	Real	0.739	0.807	0.902	0.694	0.110	0.691	0.769	0.786	0.878
	All	0.683	0.787	0.872	0.146	0.331	0.455	0.663	0.766	0.832
RMSE	Bmock12	29.62	14.17	14.43	14.34	11.67	7.68	26.12	13.14	13.55
	MBARK26	10.73	12.28	7.48	10.05	11.44	8.20	11.29	12.76	9.11
	Rinke	15.01	17.33	10.59	3.32	17.56	9.41	11.55	16.08	9.87
	ZymoCS	5.78	12.41	11.18	1.29	14.50	8.18	5.45	16.48	16.75
	Quince	22.54	17.92	14.68	30.61	23.90	23.85	23.46	18.04	16.32
	Real	17.51	15.07	10.74	8.63	14.71	8.67	15.20	14.66	11.08
	All	20.14	16.53	12.83	22.33	19.77	17.83	19.70	16.41	13.91

[a] R_{lm}^2 : fraction of explained variance after performing a linear fit; $R_{x\sim y}^2$: fraction of explained variance of the raw prediction; RMSE: root mean square error.

Supplementary Table 8. Median absolute difference between predicted values for Proteobacteria bins in the test set, and the same bins to with the addition of the complete sequence of a plasmid

Plasmid	Metric	CheckM	MAGISTA	MAGISTIC
piPO2T	Completeness	0	0.75	0.30
	“Purity”/Purity	0	1.23	0.84
RK2	Completeness	0	1.42	0.71
	“Purity”/Purity	0	3.64	2.09
R388	Completeness	0	0.96	0.41
	“Purity”/Purity	0	1.56	1.44
