# Supplementary material for: Accurate prediction of metagenome-assembled genome completeness by MAGISTA, a random forest model built on alignment-free intra-bin statistics
Source: Environ Microbiome. 2022 Mar 5;17:9. doi: 10.1186/s40793-022-00403-7 (PMC8898458; doi:10.1186/s40793-022-00403-7)
Supplement: Supplementary file 3 — Additional file 3. Graphical comparison of the taxonomy, length, and GC content of all genomes included in the HC227 mock. [file 40793_2022_403_MOESM3_ESM.pdf]

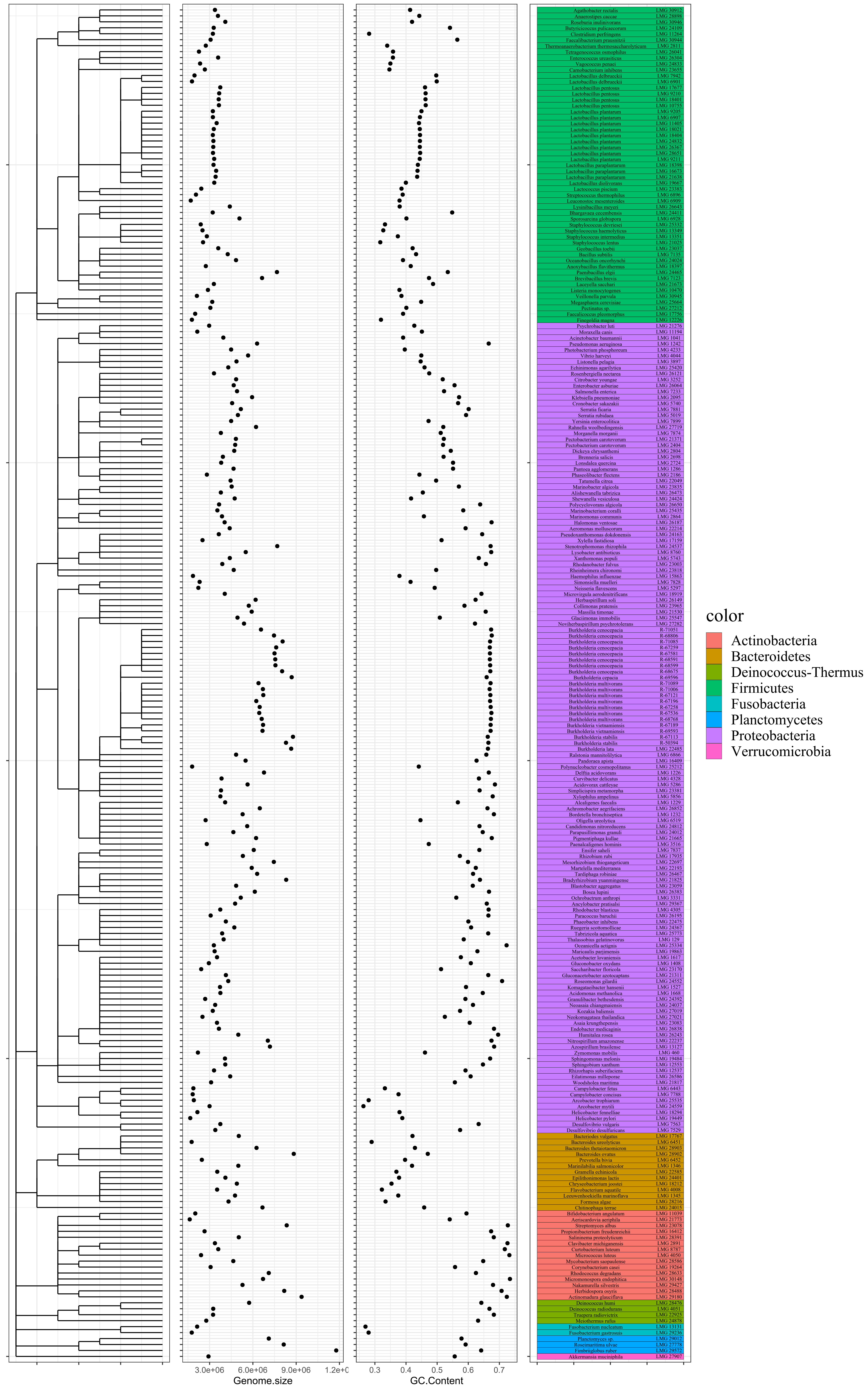

Graphical comparison of the reference assemblies for HC277, including taxonomic classification, genome size, GC content and Phylum (color).
