# Supplementary material for: Accurate prediction of metagenome-assembled genome completeness by MAGISTA, a random forest model built on alignment-free intra-bin statistics
Source: Environ Microbiome. 2022 Mar 5;17:9. doi: 10.1186/s40793-022-00403-7 (PMC8898458; doi:10.1186/s40793-022-00403-7)
Supplement: Supplementary file 4 — Additional file 4. Closest analogues for completeness and purity obtained from the output of CheckM (a, b) and GUNC (c, d) as a function of the actual values in the training dataset bins. Data points are coloured according to the binner used. The blue line is the best linear fit. [file 40793_2022_403_MOESM4_ESM.pdf]

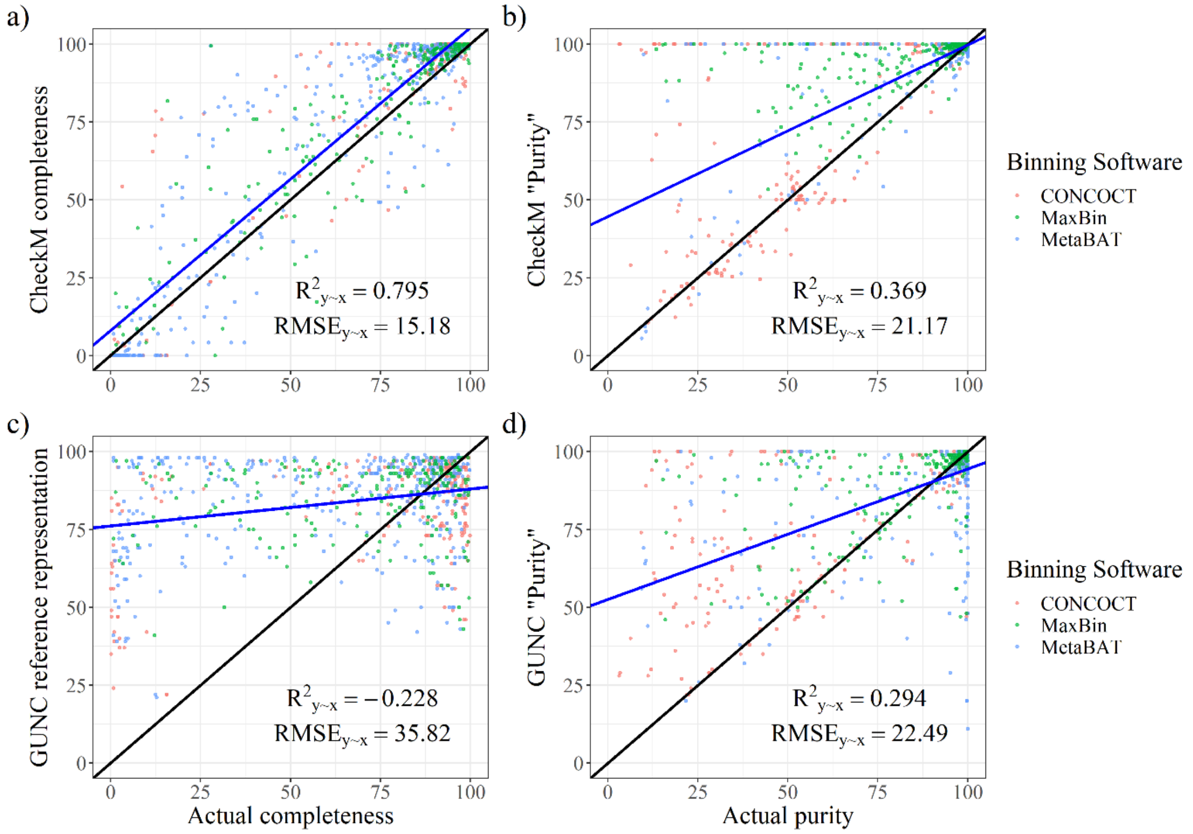

Closest analogues for completeness and purity obtained from the output of CheckM (a,b) and GUNC (c,d) as a function of the actual values in the training dataset bins. Data points are coloured according to the binner used. The blue line is the best linear fit.
