# Supplementary material for: Accurate prediction of metagenome-assembled genome completeness by MAGISTA, a random forest model built on alignment-free intra-bin statistics
Source: Environ Microbiome. 2022 Mar 5;17:9. doi: 10.1186/s40793-022-00403-7 (PMC8898458; doi:10.1186/s40793-022-00403-7)
Supplement: Supplementary file 5 — Additional file 5. Cross validation prediction results for HC277. [file 40793_2022_403_MOESM5_ESM.pdf]

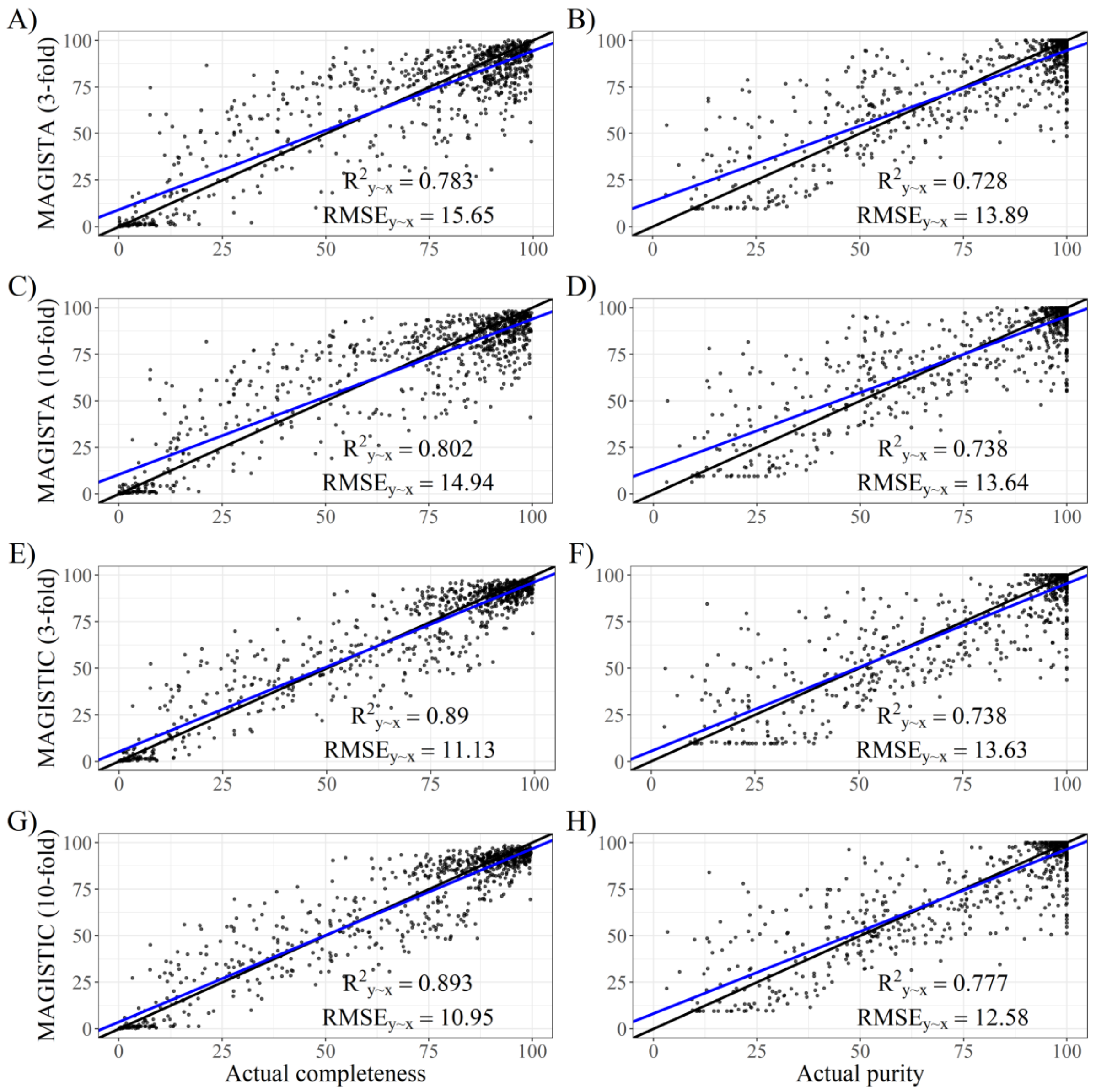

Cross validation prediction results for HC277. All sub-figures show a predicted statistic according to the expected (real) value, and provide an evaluation of the expected performance of the model using R2 and RMSE values. Columns correspond to different predicted statistics: Completeness (A,C,E,G) and Purity (B,D,F,H); while rows correspond to a different cross-validation analysis: MAGISTA, using 3-fold cross-validation (A,B) or 10-fold cross-validation (C,D) and MAGISTIC, using using 3-fold cross-validation (E,F) or 10-fold cross-validation (G,H). These plots have been added to illustrate that cross-validation is not ideal for predicting the model's performance, shown in Figure 7 of the main text.
