# Supplementary material for: Accurate prediction of metagenome-assembled genome completeness by MAGISTA, a random forest model built on alignment-free intra-bin statistics
Source: Environ Microbiome. 2022 Mar 5;17:9. doi: 10.1186/s40793-022-00403-7 (PMC8898458; doi:10.1186/s40793-022-00403-7)
Supplement: Supplementary file 6 — Additional file 6. Variants of Fig. 7 showing the effect of taxonomic distance from the reference or training set on the performance of the model. For each bin, its taxonomic distance from the reference or training set is defined as the taxonomic difference between its best matching genome (see materials and methods) and the closest genome in the reference or training dataset. Each sub-figure corresponds to a “target distance” (i.e. same species, genus, family, order, class or phylum). The six leftmost plots contain only the bins whose distance is exactly the target distance, the six middle plots contain all bins whose taxonomic distance is less than or equal to the target distance, and the six rightmost plots contain only the bins with a taxonomic distance to the reference or training dataset above the target distance. [file 40793_2022_403_MOESM6_ESM.pdf]

Additional File 6 – Variants of Figure 7 showing the effect of taxonomic distance from the reference or training set on the performance of the model. For each bin, its taxonomic distance from the reference or training set is defined as the taxonomic difference between its best matching genome (see materials and methods) and the closest genome in the reference or training dataset. Each sub-figure corresponds to a “target distance” (i.e. same species, genus, family, order, class or phylum). The six leftmost plots contain only the bins whose distance is exactly the target distance, the six middle plots contain all bins whose taxonomic distance is less than or equal to the target distance, and the six rightmost plots contain only the bins with a taxonomic distance to the reference or training dataset above the target distance.

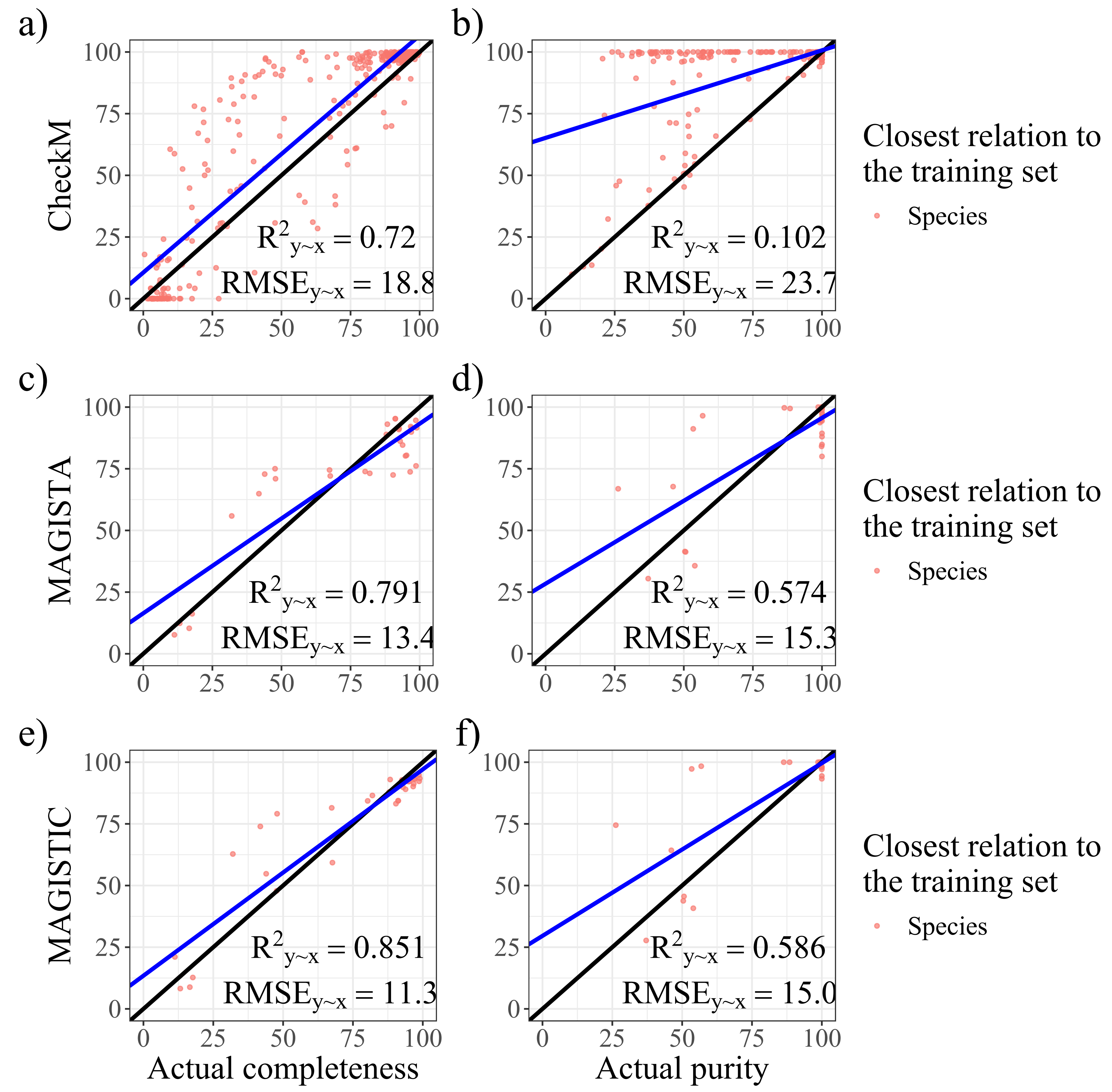
a)
b)
c)
d)
e)
f)
CheckM
MAGISTA
MAGISTIC
R2y~x = 0.72
RMSEy~x = 18.8
R2y~x = 0.102
RMSEy~x = 23.7
R2y~x = 0.791
RMSEy~x = 13.4
R2y~x = 0.574
RMSEy~x = 15.3
R2y~x = 0.851
RMSEy~x = 11.3
R2y~x = 0.586
RMSEy~x = 15.0
Closest relation to the training set
Species
Actual completeness
Actual purity

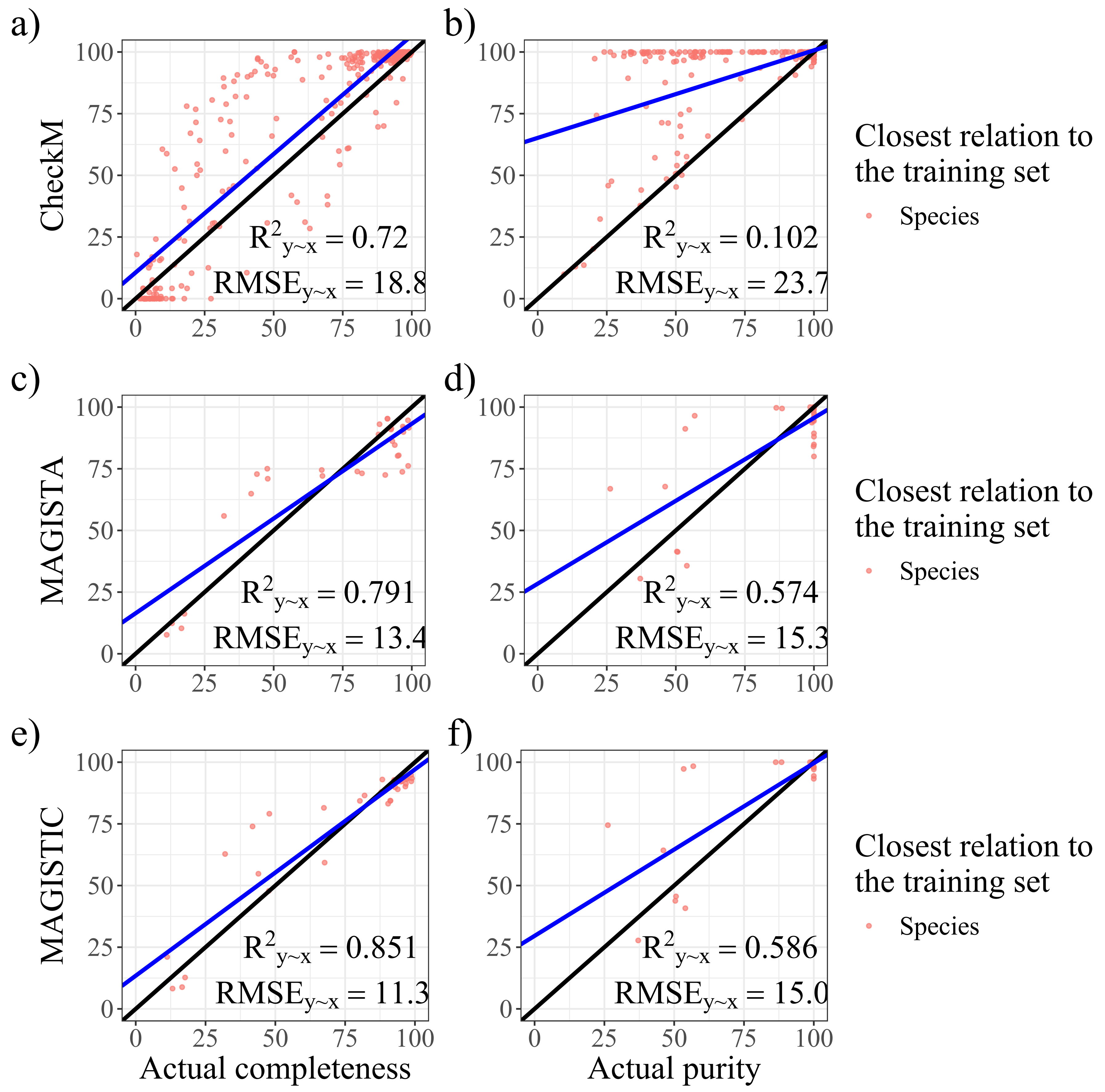
a)
b)
c)
d)
e)
f)
CheckM
MAGISTA
MAGISTIC
R2y~x = 0.72
RMSEy~x = 18.8
R2y~x = 0.102
RMSEy~x = 23.7
R2y~x = 0.791
RMSEy~x = 13.4
R2y~x = 0.574
RMSEy~x = 15.3
R2y~x = 0.851
RMSEy~x = 11.3
R2y~x = 0.586
RMSEy~x = 15.0
Closest relation to the training set
Species
Actual completeness
Actual purity

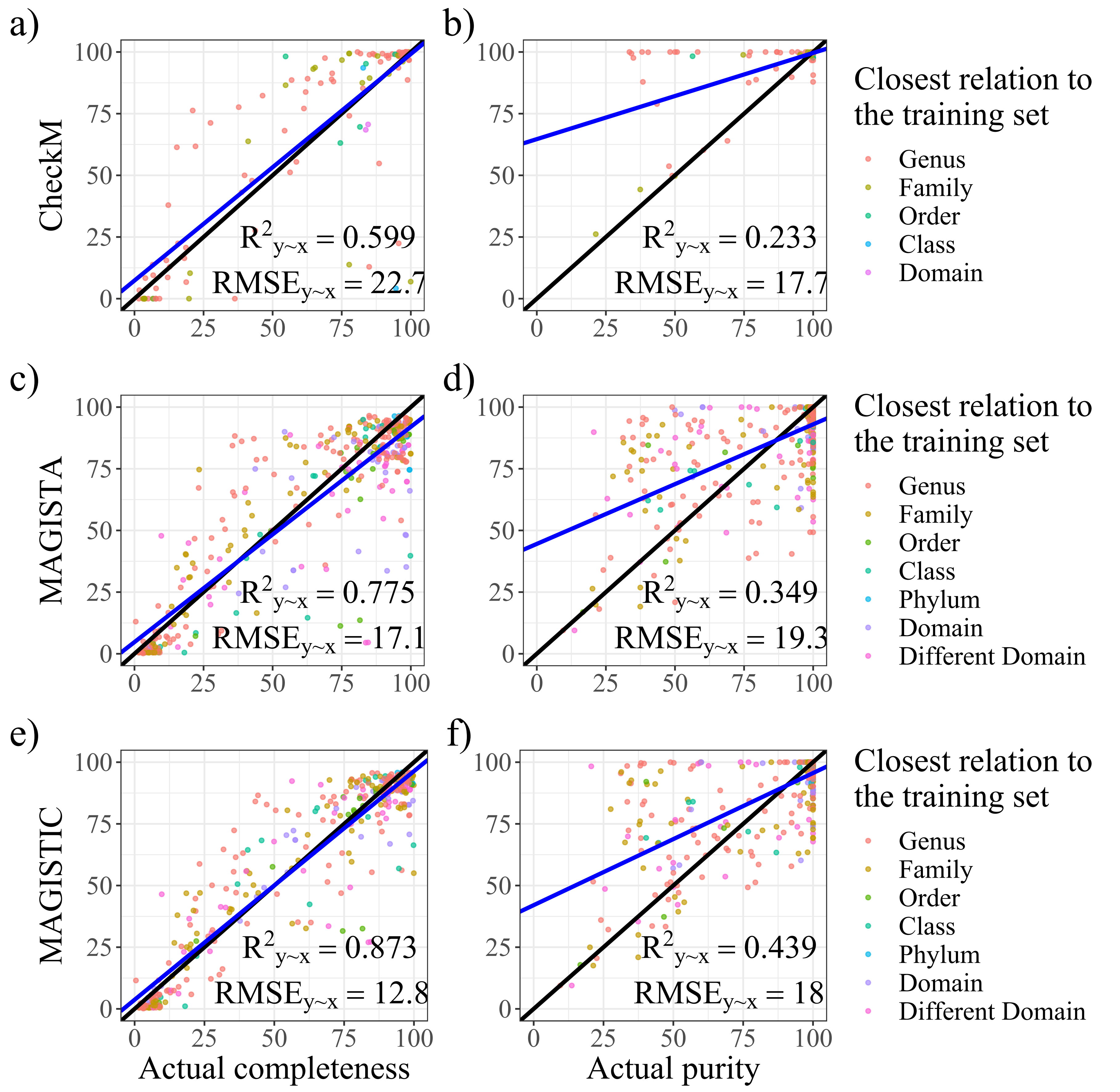
a)
b)
c)
d)
e)
f)
CheckM
MAGISTA
MAGISTIC
R2y~x = 0.599
RMSEy~x = 22.7
R2y~x = 0.233
RMSEy~x = 17.7
R2y~x = 0.775
RMSEy~x = 17.1
R2y~x = 0.349
RMSEy~x = 19.3
R2y~x = 0.873
RMSEy~x = 12.8
R2y~x = 0.439
RMSEy~x = 18
Closest relation to the training set
Genus
Family
Order
Class
Phylum
Domain
Different Domain
Actual completeness
Actual purity

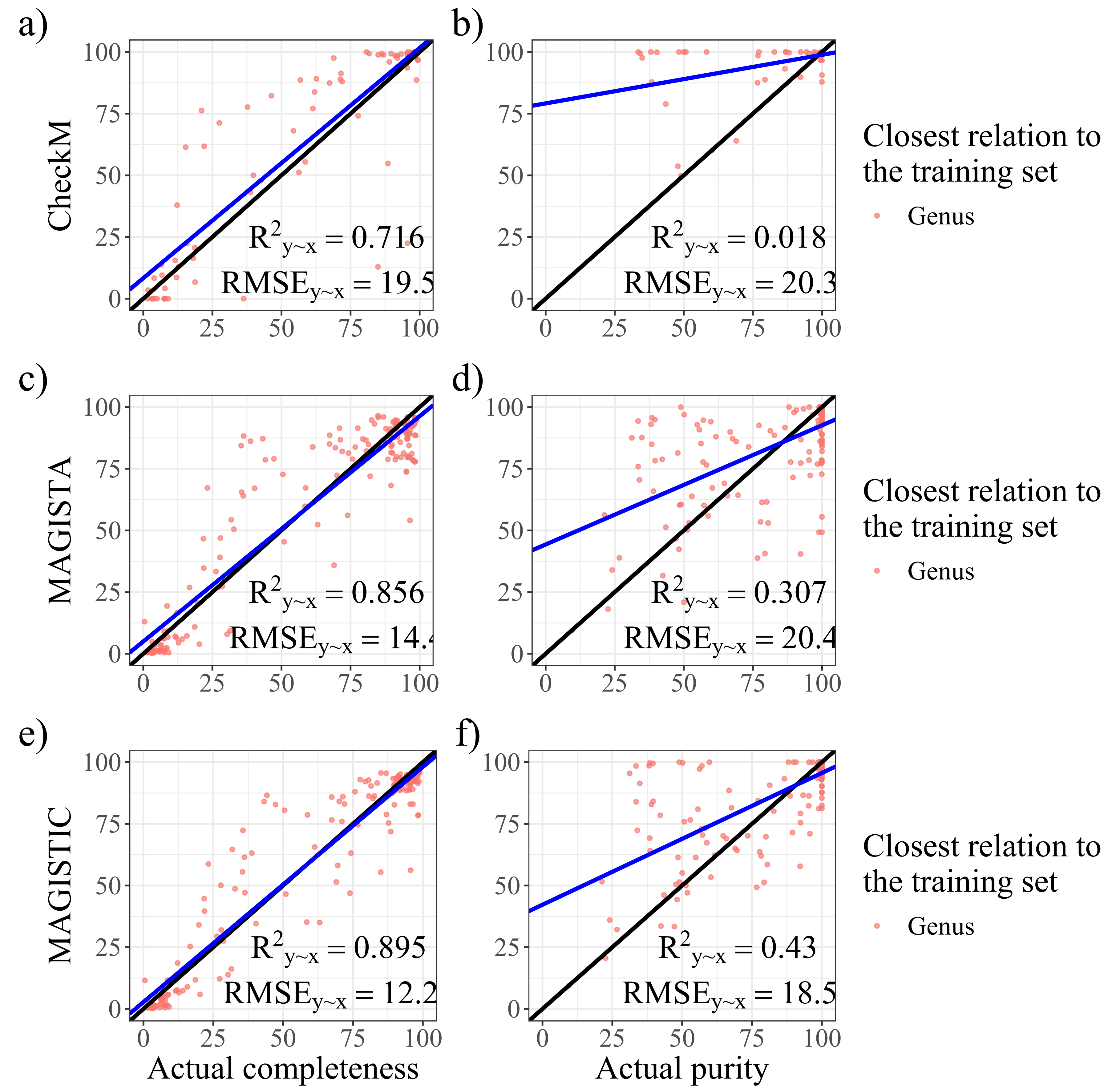
a)
b)
c)
d)
e)
f)
CheckM
MAGISTA
MAGISTIC
R2y~x = 0.716
RMSEy~x = 19.5
R2y~x = 0.018
RMSEy~x = 20.3
R2y~x = 0.856
RMSEy~x = 14.4
R2y~x = 0.307
RMSEy~x = 20.4
R2y~x = 0.895
RMSEy~x = 12.2
R2y~x = 0.43
RMSEy~x = 18.5
Closest relation to the training set
Genus
Actual completeness
Actual purity

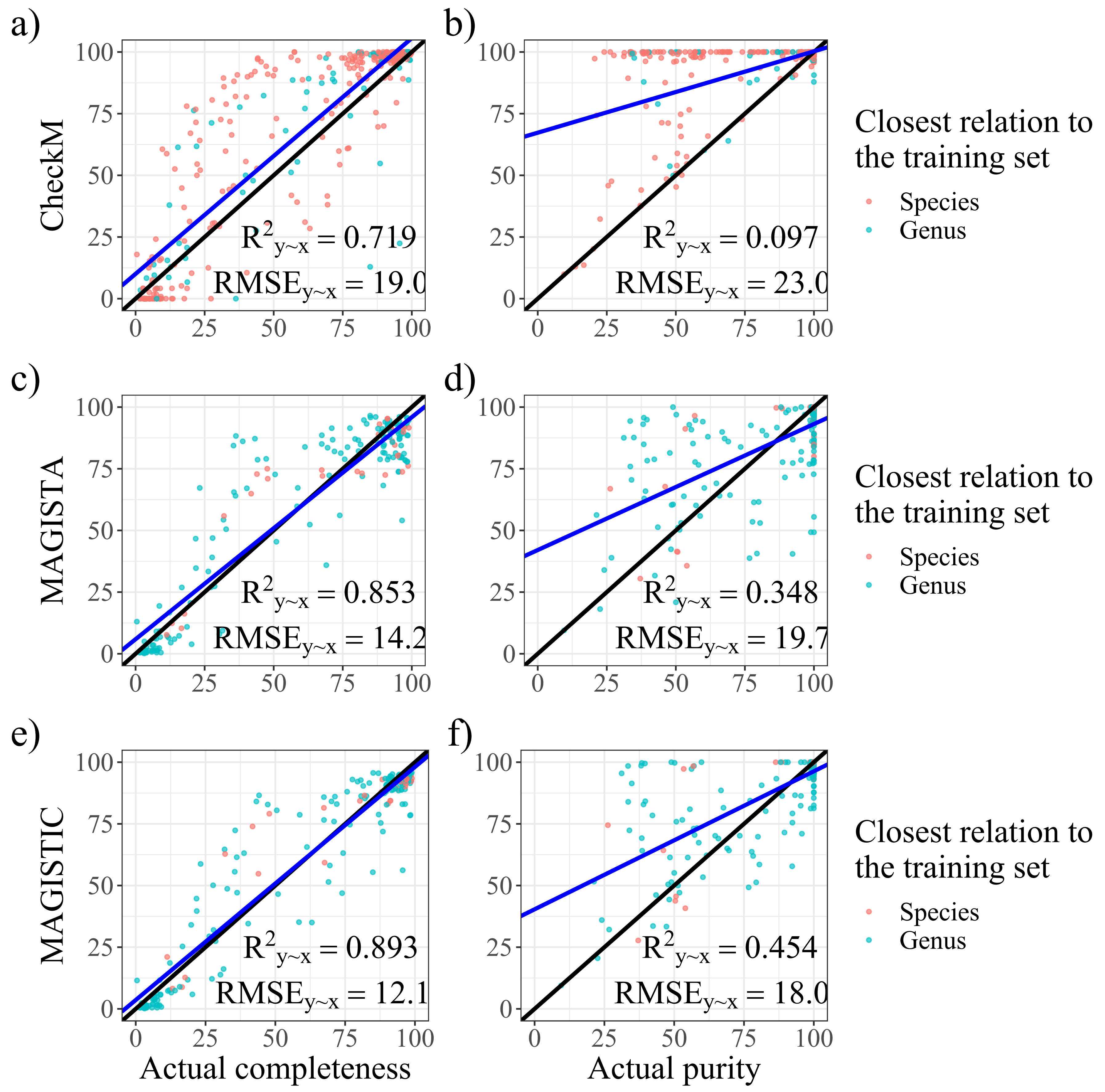
a)
b)
c)
d)
e)
f)
CheckM
MAGISTA
MAGISTIC
R2y~x = 0.719
RMSEy~x = 19.0
R2y~x = 0.097
RMSEy~x = 23.0
R2y~x = 0.853
RMSEy~x = 14.2
R2y~x = 0.348
RMSEy~x = 19.7
R2y~x = 0.893
RMSEy~x = 12.1
R2y~x = 0.454
RMSEy~x = 18.0
Closest relation to the training set
Species
Genus
Actual completeness
Actual purity

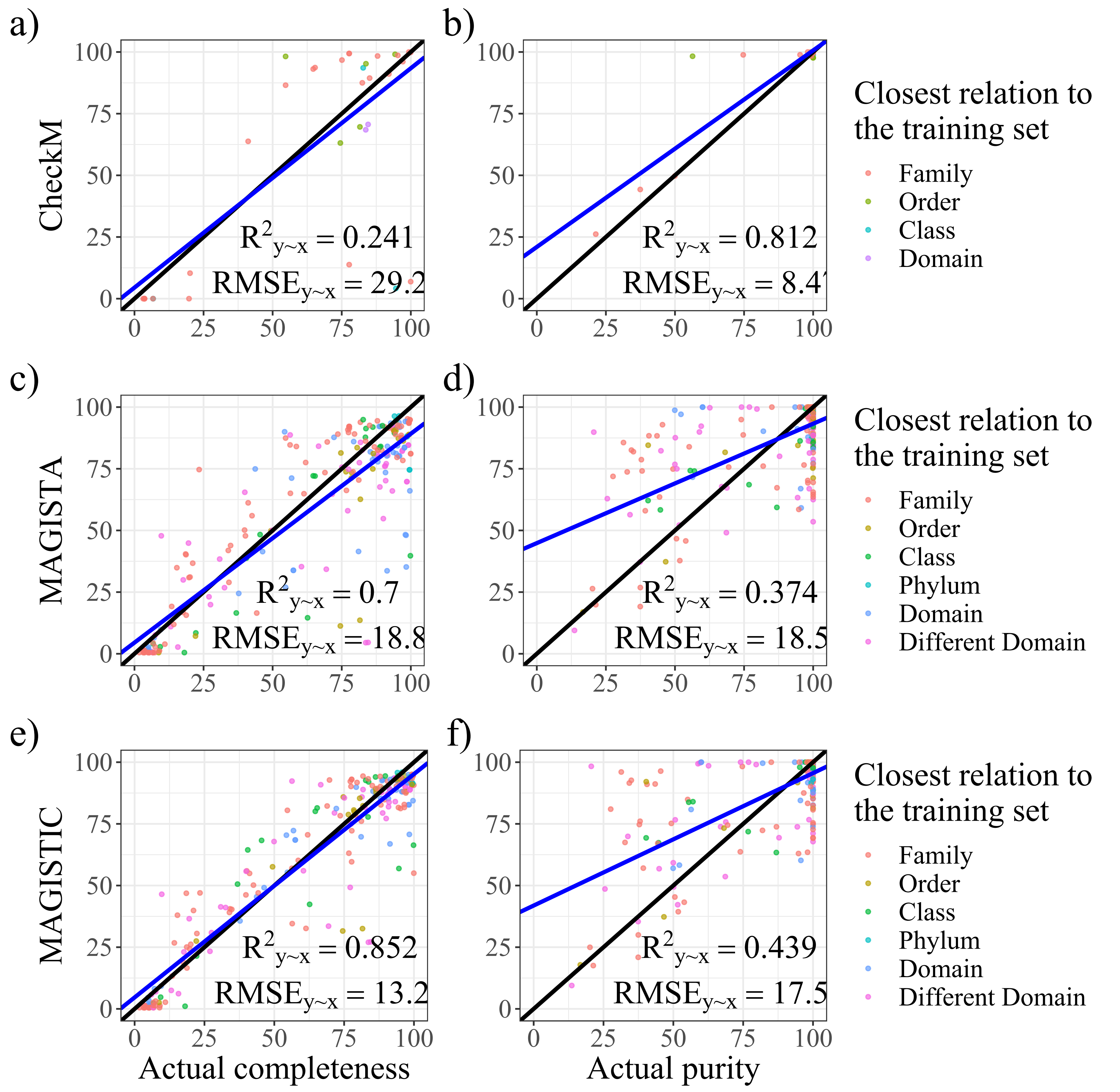
a)
b)
c)
d)
e)
f)
CheckM
MAGISTA
MAGISTIC
R2y~x = 0.241
RMSEy~x = 29.2
R2y~x = 0.812
RMSEy~x = 8.41
R2y~x = 0.7
RMSEy~x = 18.8
R2y~x = 0.374
RMSEy~x = 18.5
R2y~x = 0.852
RMSEy~x = 13.2
R2y~x = 0.439
RMSEy~x = 17.5
Closest relation to the training set
Family
Order
Class
Phylum
Domain
Different Domain
Actual completeness
Actual purity

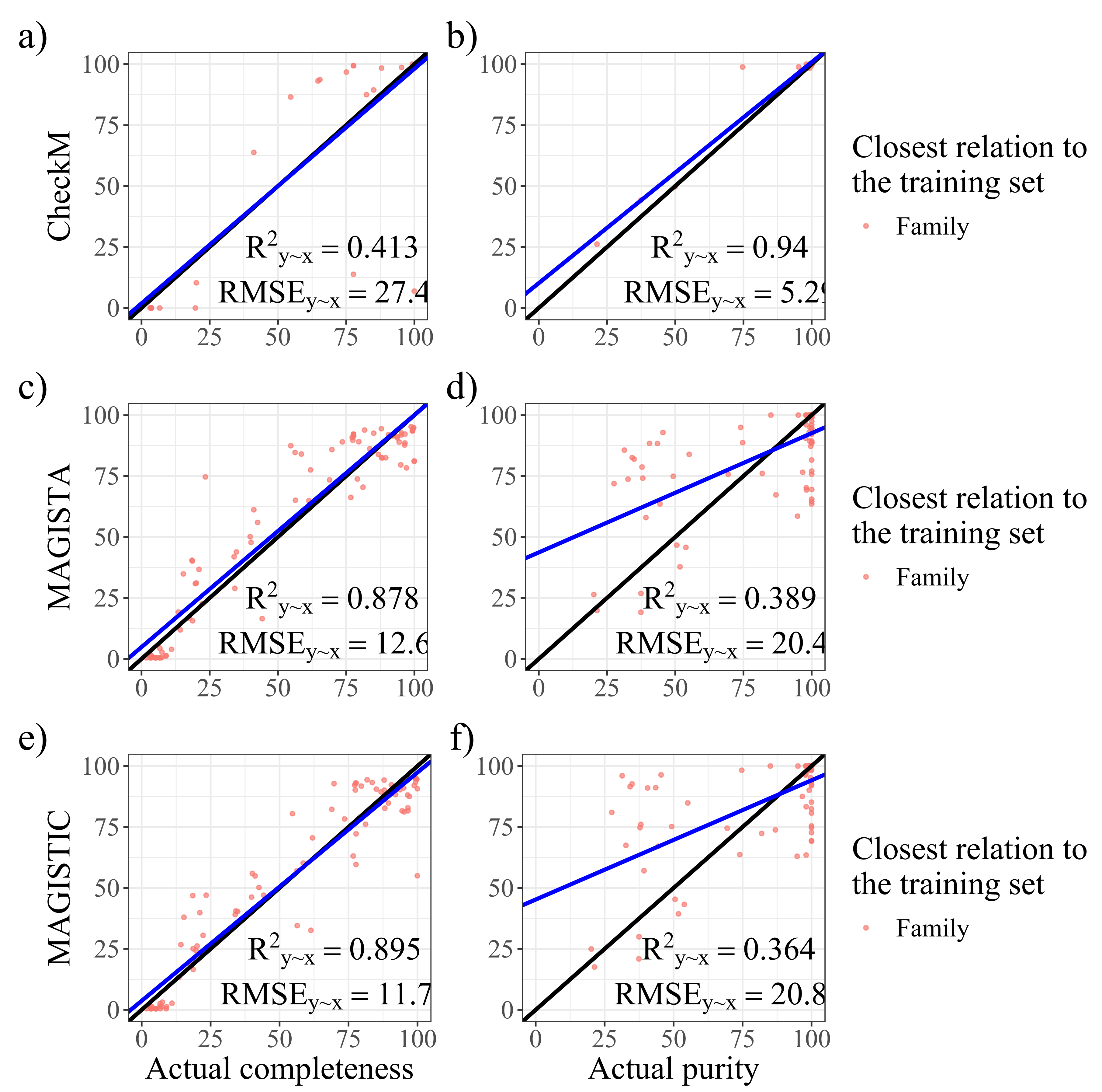

a)
b)
c)
d)
e)
f)
CheckM
MAGISTA
MAGISTIC
R2y~x = 0.413
RMSEy~x = 27.4
R2y~x = 0.94
RMSEy~x = 5.2
R2y~x = 0.878
RMSEy~x = 12.6
R2y~x = 0.389
RMSEy~x = 20.4
R2y~x = 0.895
RMSEy~x = 11.7
R2y~x = 0.364
RMSEy~x = 20.8
Closest relation to the training set
Family
Actual completeness
Actual purity

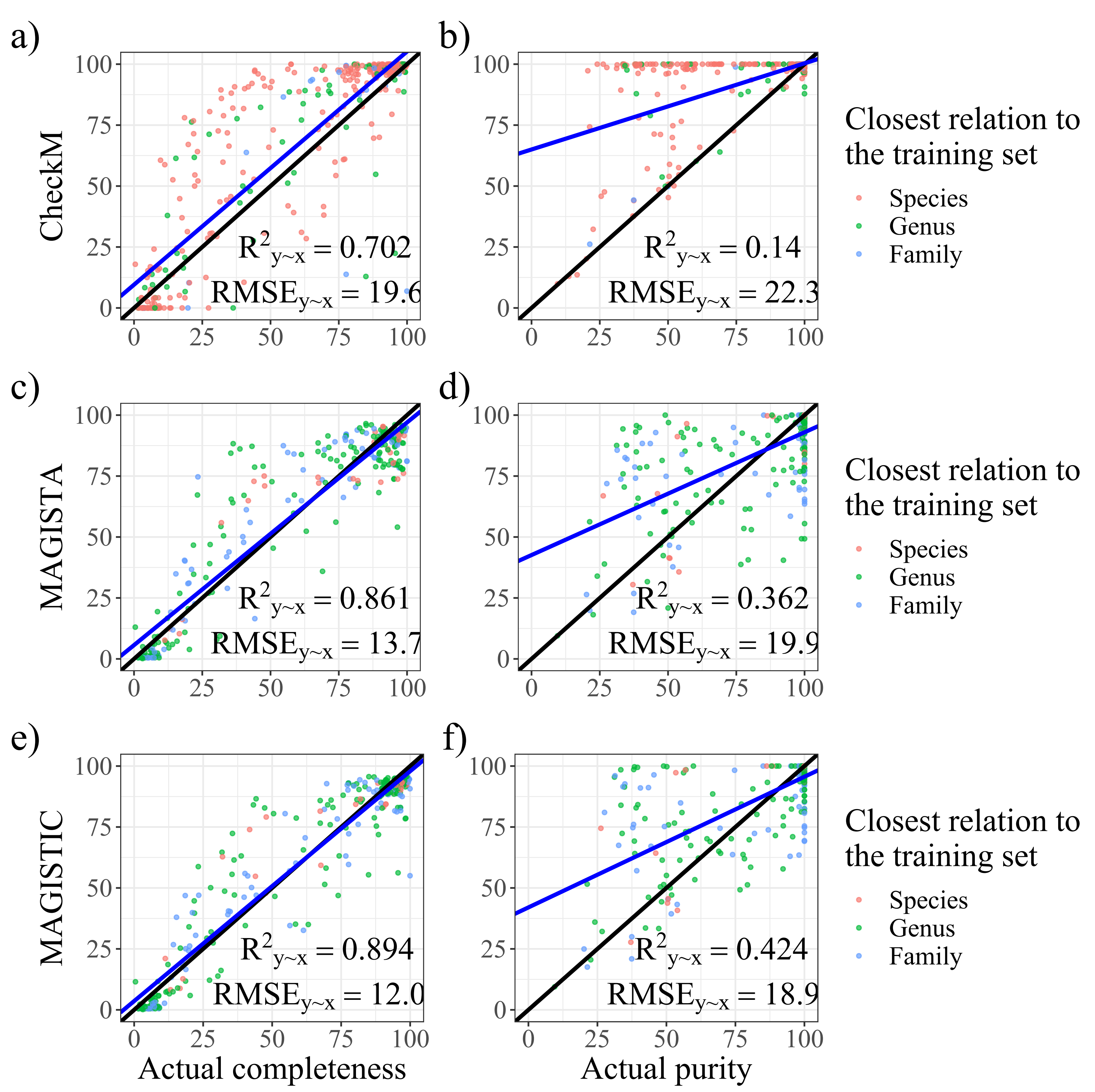

a)
b)
c)
d)
e)
f)
CheckM
MAGISTA
MAGISTIC
R2y~x = 0.702
RMSEy~x = 19.6
R2y~x = 0.14
RMSEy~x = 22.3
R2y~x = 0.861
RMSEy~x = 13.7
R2y~x = 0.362
RMSEy~x = 19.9
R2y~x = 0.894
RMSEy~x = 12.0
R2y~x = 0.424
RMSEy~x = 18.9
Closest relation to the training set
Species
Genus
Family
Actual completeness
Actual purity

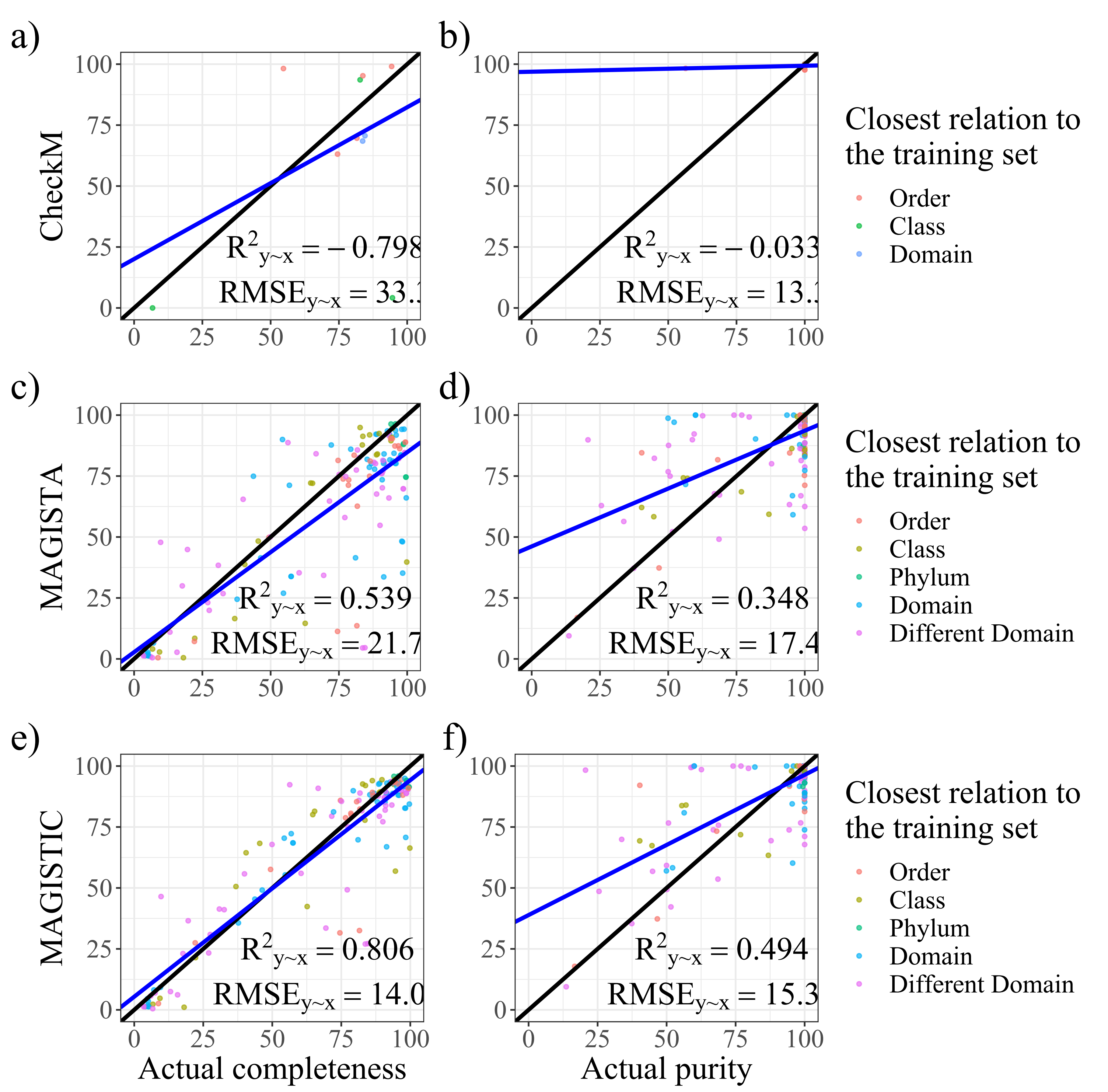

a)
b)
c)
d)
e)
f)
CheckM
MAGISTA
MAGISTIC
R2y~x = −0.798
RMSEy~x = 33.
R2y~x = −0.033
RMSEy~x = 13.
R2y~x = 0.539
RMSEy~x = 21.7
R2y~x = 0.348
RMSEy~x = 17.4
R2y~x = 0.806
RMSEy~x = 14.0
R2y~x = 0.494
RMSEy~x = 15.3
Closest relation to the training set
Order
Class
Phylum
Domain
Different Domain
Actual completeness
Actual purity

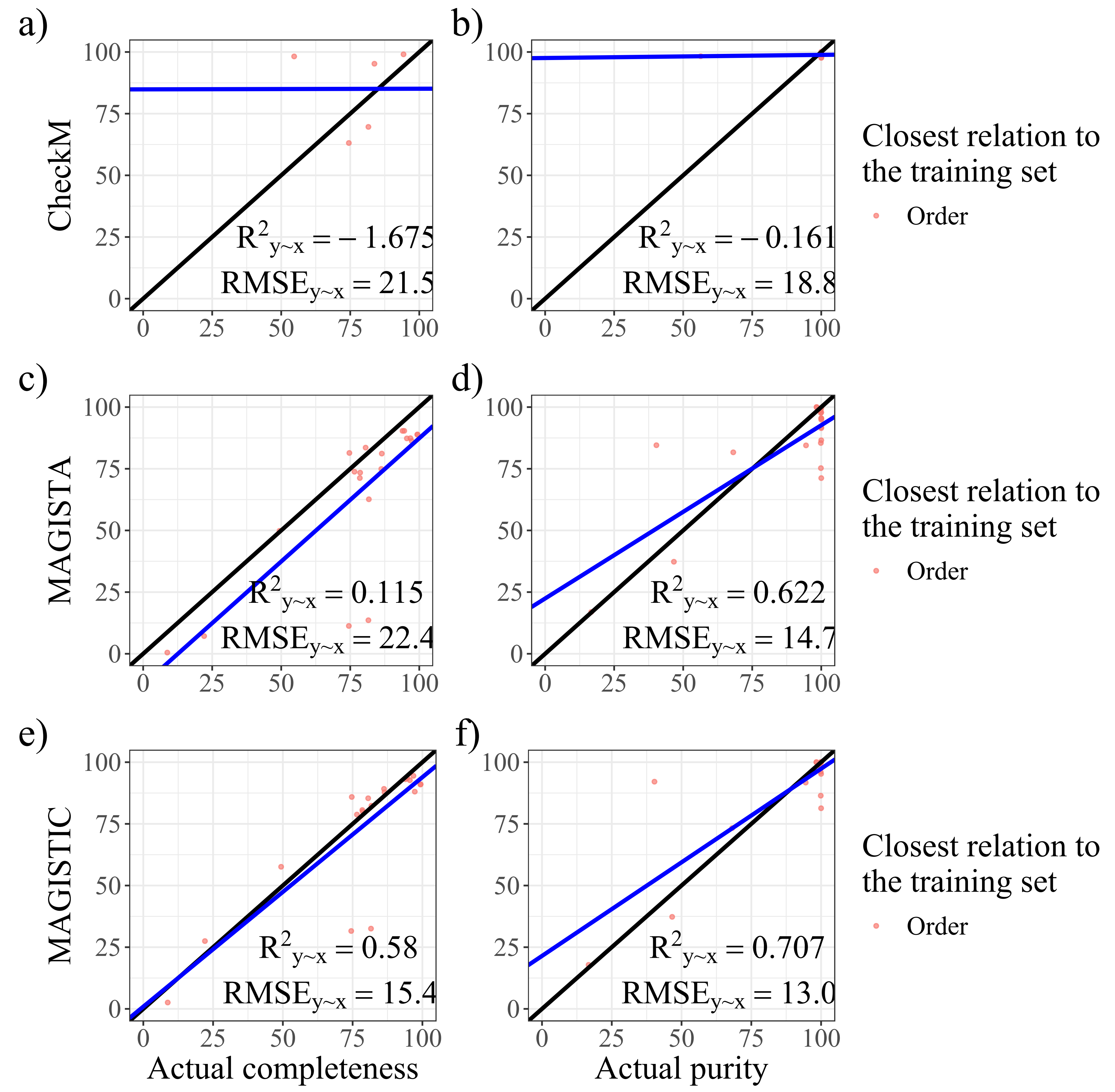
a)
b)
c)
d)
e)
f)
CheckM
MAGISTA
MAGISTIC
R2y~x = −1.675
RMSEy~x = 21.5
R2y~x = −0.161
RMSEy~x = 18.8
R2y~x = 0.115
RMSEy~x = 22.4
R2y~x = 0.622
RMSEy~x = 14.7
R2y~x = 0.58
RMSEy~x = 15.4
R2y~x = 0.707
RMSEy~x = 13.0
Closest relation to the training set
Order
Actual completeness
Actual purity

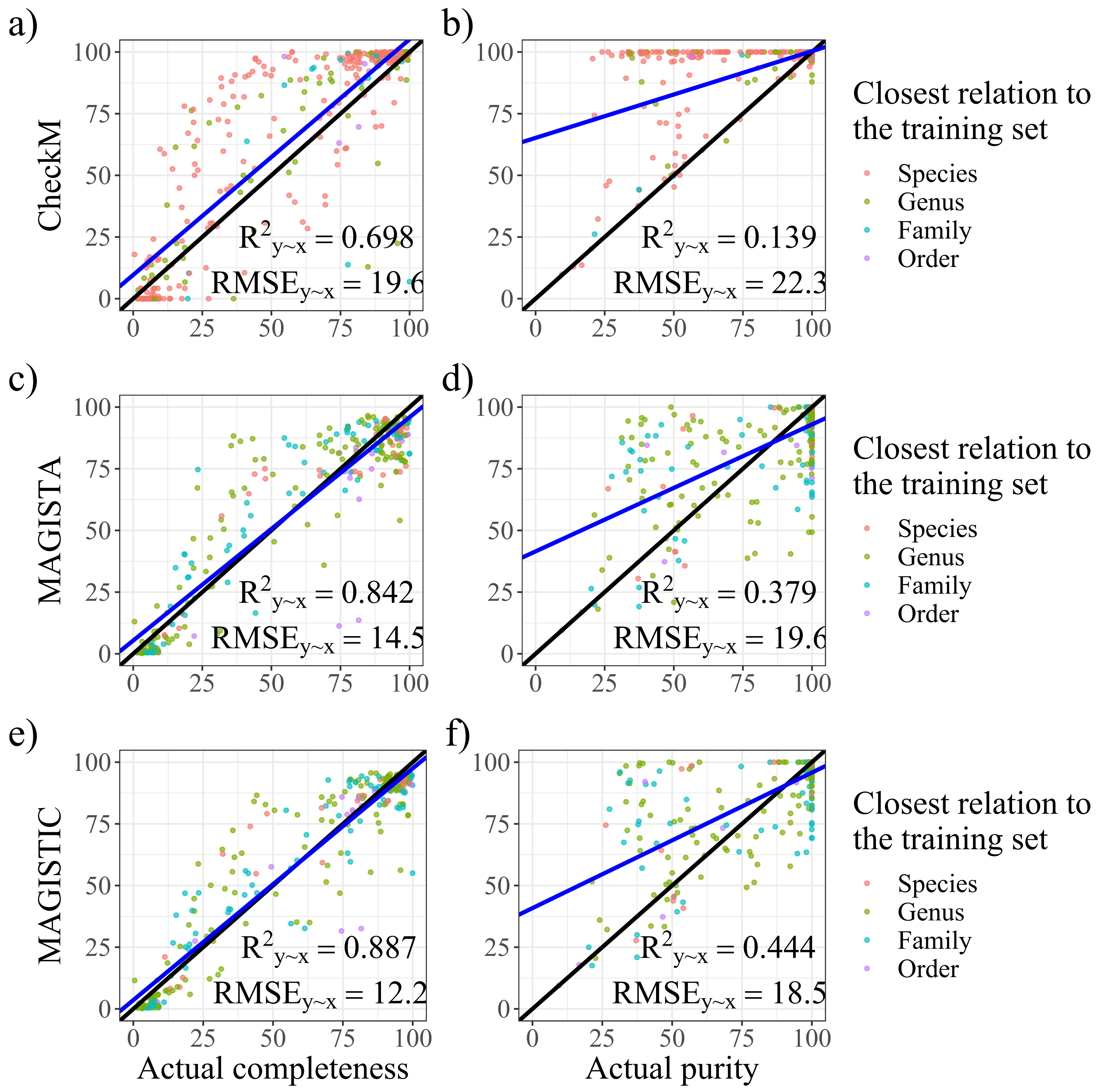
a)
b)
c)
d)
e)
f)
CheckM
MAGISTA
MAGISTIC
R2y~x = 0.698
RMSEy~x = 19.6
R2y~x = 0.139
RMSEy~x = 22.3
R2y~x = 0.842
RMSEy~x = 14.5
R2y~x = 0.379
RMSEy~x = 19.6
R2y~x = 0.887
RMSEy~x = 12.2
R2y~x = 0.444
RMSEy~x = 18.5
Closest relation to the training set
Species
Genus
Family
Order
Actual completeness
Actual purity

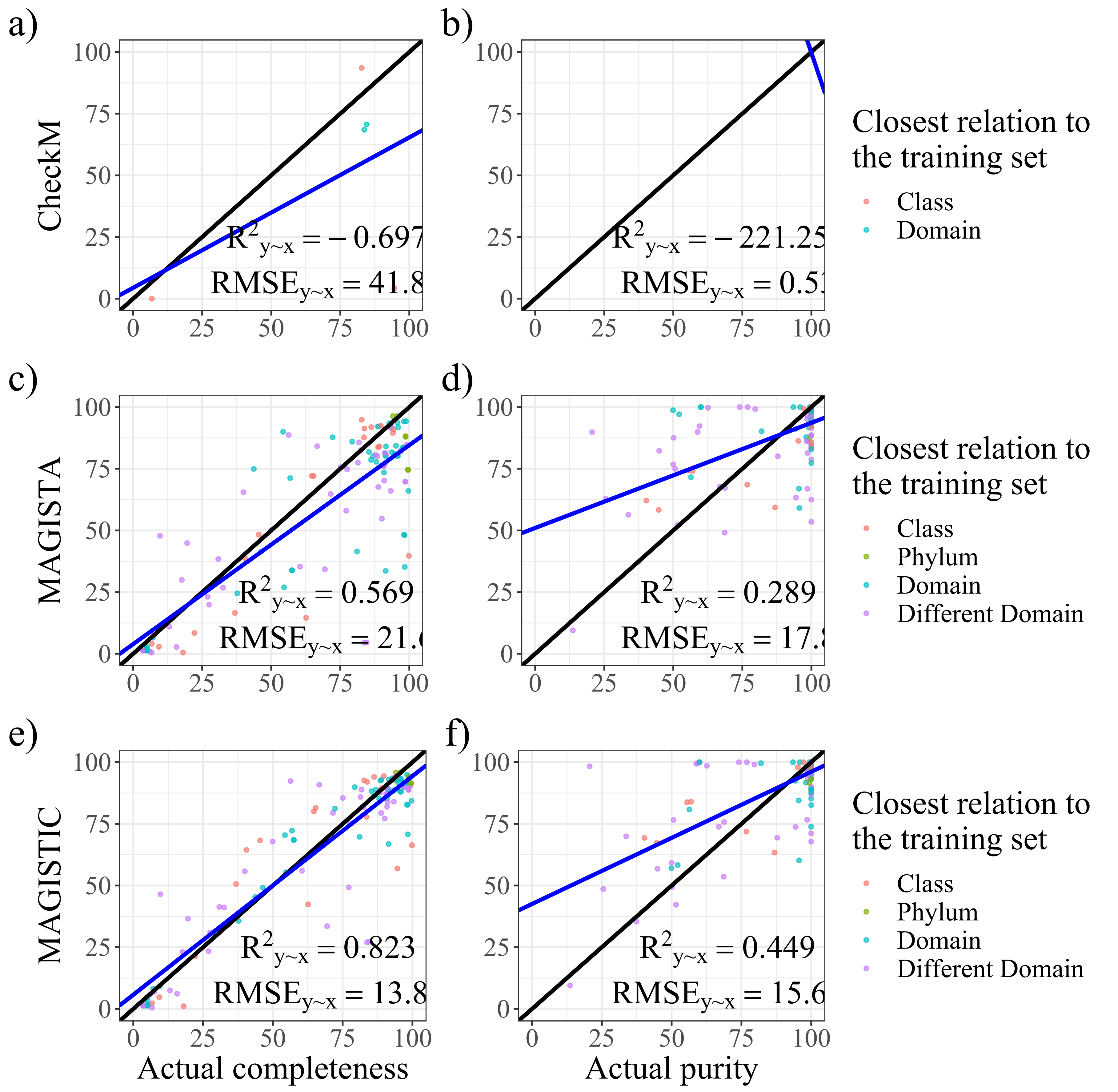
a)
b)
c)
d)
e)
f)
CheckM
MAGISTA
MAGISTIC
R2y~x = −0.697
RMSEy~x = 41.8
R2y~x = −221.25
RMSEy~x = 0.5
R2y~x = 0.569
RMSEy~x = 21.
R2y~x = 0.289
RMSEy~x = 17.
R2y~x = 0.823
RMSEy~x = 13.8
R2y~x = 0.449
RMSEy~x = 15.6
Closest relation to the training set
Class
Domain
Phylum
Different Domain
Actual completeness
Actual purity

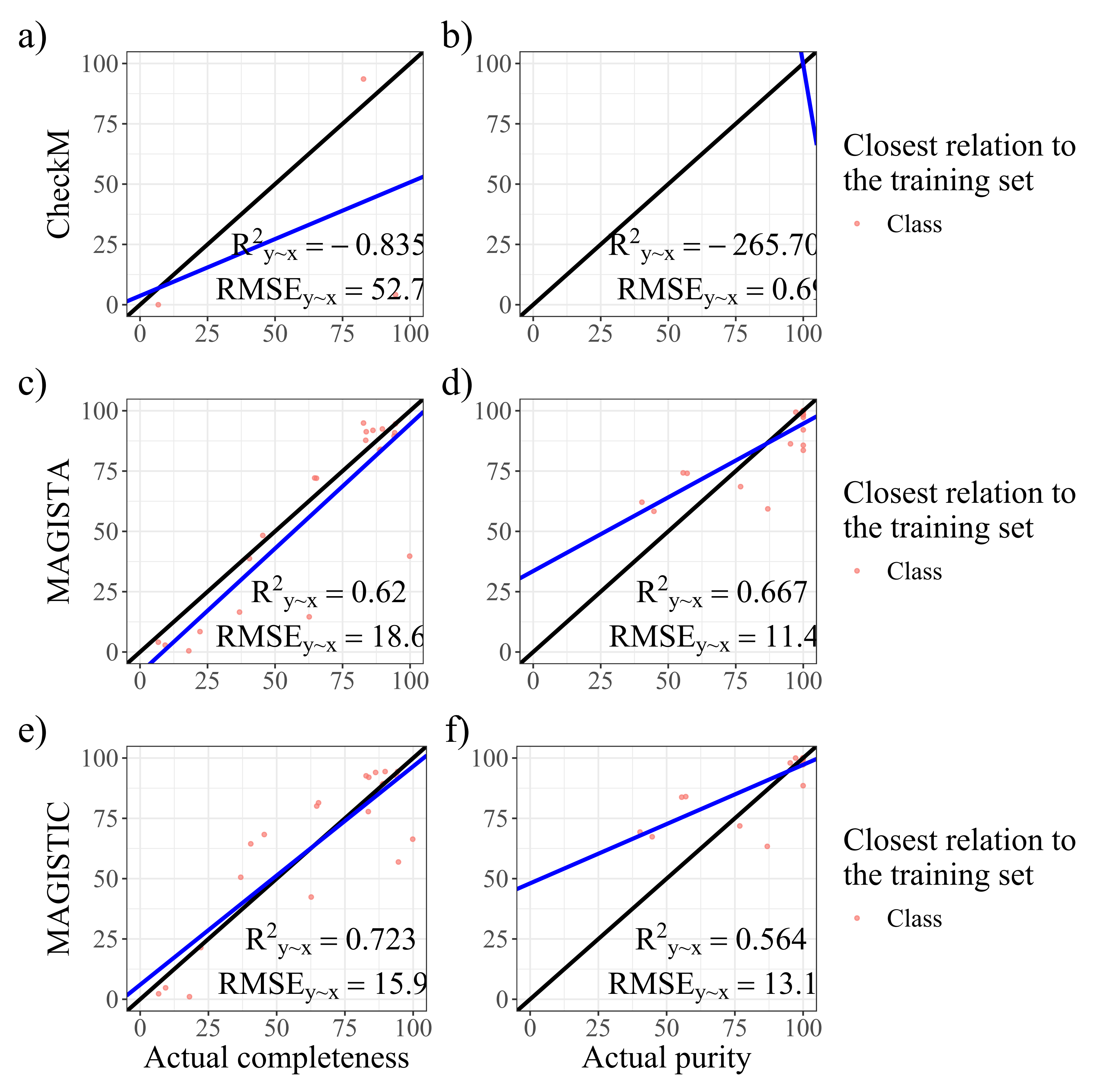

a)
b)
c)
d)
e)
f)
CheckM
MAGISTA
MAGISTIC
R2y~x = −0.835
RMSEy~x = 52.7
R2y~x = −265.70
RMSEy~x = 0.69
R2y~x = 0.62
RMSEy~x = 18.6
R2y~x = 0.667
RMSEy~x = 11.4
R2y~x = 0.723
RMSEy~x = 15.9
R2y~x = 0.564
RMSEy~x = 13.1
Closest relation to the training set
Class
Actual completeness
Actual purity

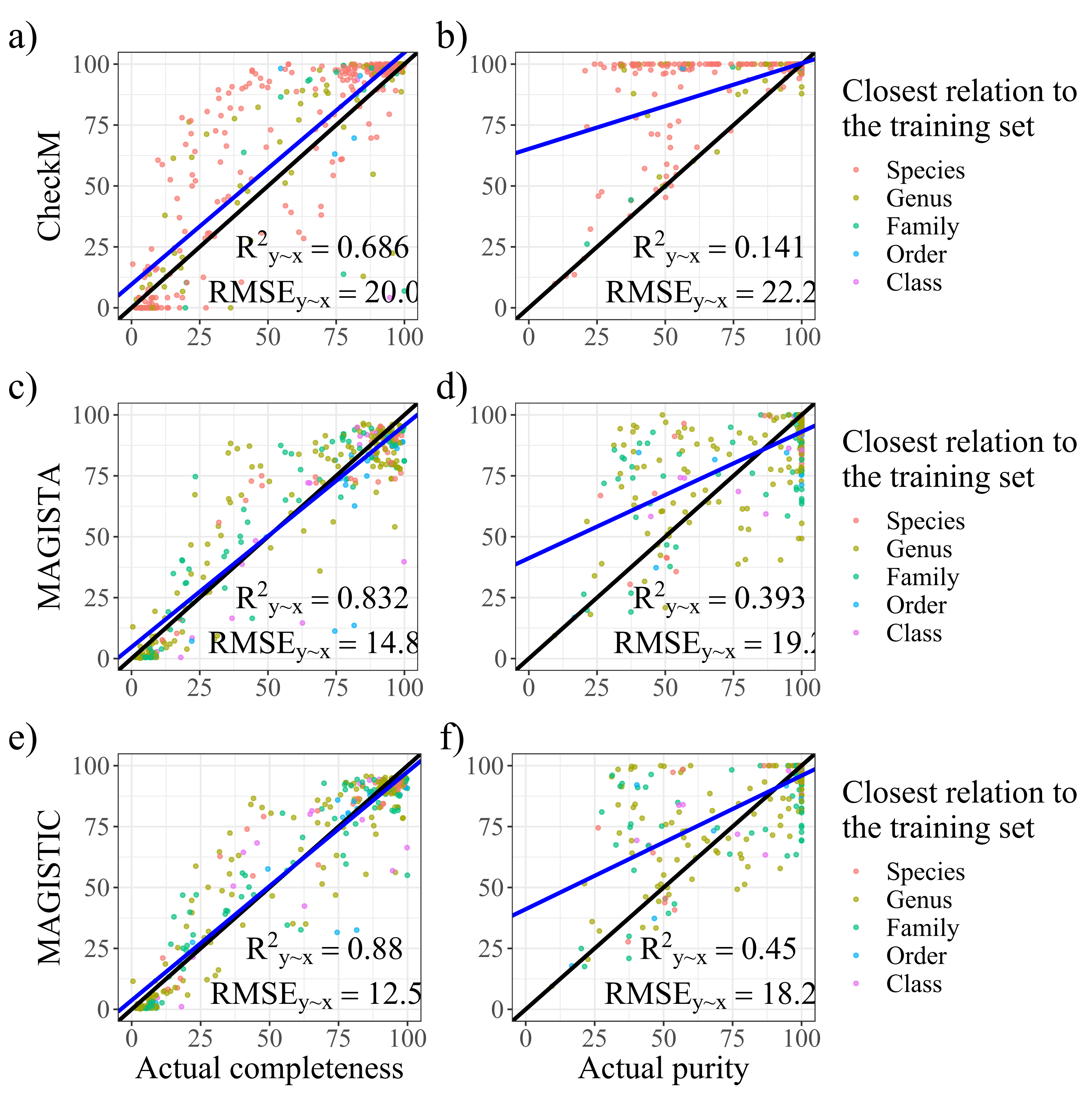

a)
b)
c)
d)
e)
f)
CheckM
MAGISTA
MAGISTIC
R2y~x = 0.686
RMSEy~x = 20.0
R2y~x = 0.141
RMSEy~x = 22.2
R2y~x = 0.832
RMSEy~x = 14.8
R2y~x = 0.393
RMSEy~x = 19.
R2y~x = 0.88
RMSEy~x = 12.5
R2y~x = 0.45
RMSEy~x = 18.2
Closest relation to the training set
Species
Genus
Family
Order
Class
Actual completeness
Actual purity

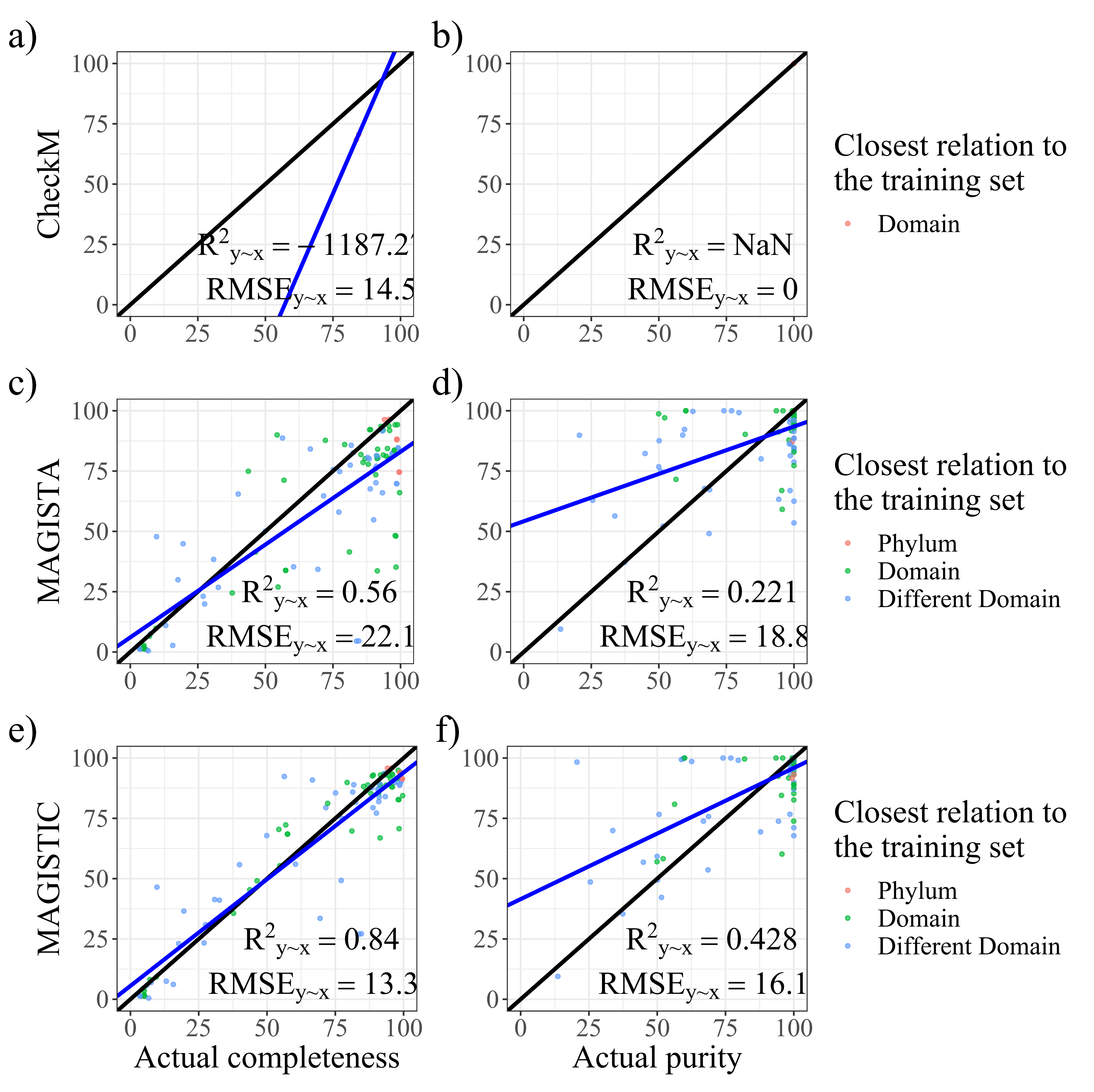

a)
b)
c)
d)
e)
f)
CheckM
MAGISTA
MAGISTIC
R2y~x = −1187.2
RMSEy~x = 14.5
R2y~x = NaN
RMSEy~x = 0
R2y~x = 0.56
RMSEy~x = 22.1
R2y~x = 0.221
RMSEy~x = 18.8
R2y~x = 0.84
RMSEy~x = 13.3
R2y~x = 0.428
RMSEy~x = 16.1
Closest relation to the training set
Domain
Phylum
Different Domain
Actual completeness
Actual purity

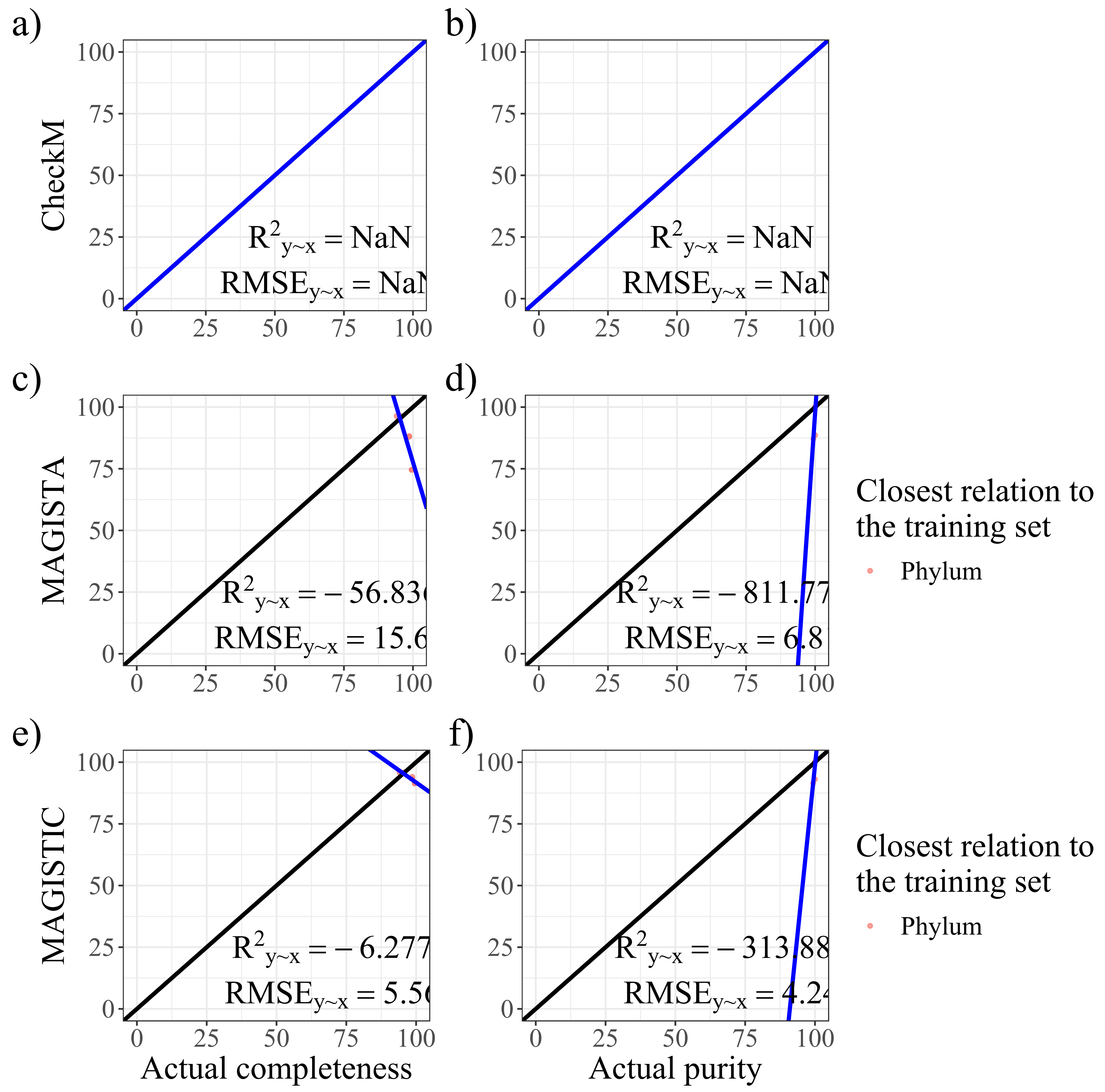
a)
b)
c)
d)
e)
f)
CheckM
MAGISTA
MAGISTIC
$R^2_{y\sim x}$ = NaN
$RMSE_{y\sim x}$ = NaN
$R^2_{y\sim x}$ = − 56.83
$RMSE_{y\sim x}$ = 15.6
$R^2_{y\sim x}$ = − 811.77
$RMSE_{y\sim x}$ = 6.8
$R^2_{y\sim x}$ = − 6.277
$RMSE_{y\sim x}$ = 5.5
$R^2_{y\sim x}$ = − 313.88
$RMSE_{y\sim x}$ = 4.2
Closest relation to the training set
Phylum
Actual completeness
Actual purity

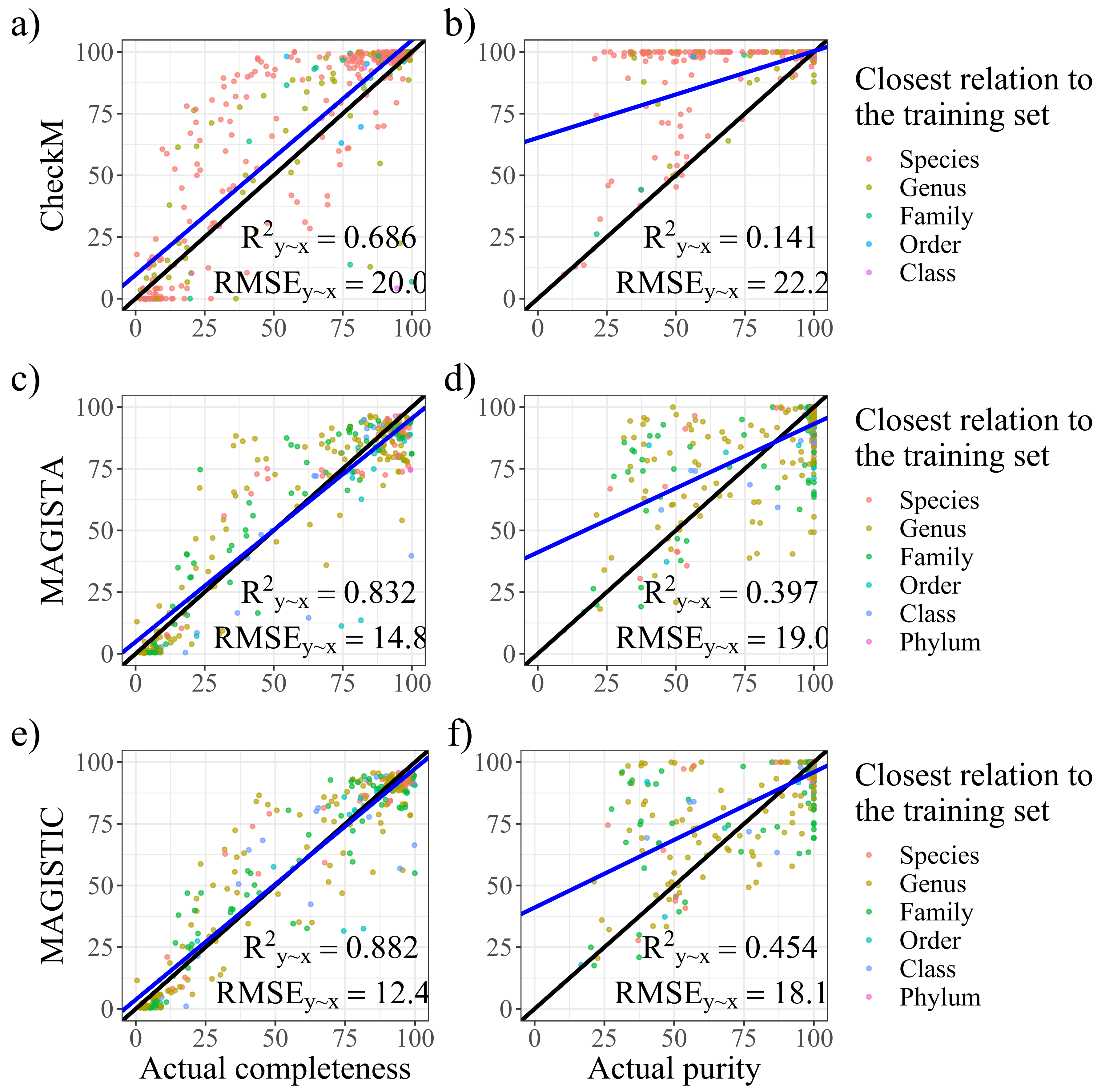
a)
b)
c)
d)
e)
f)
CheckM
MAGISTA
MAGISTIC
$R^2_{y\sim x}$ = 0.686
$RMSE_{y\sim x}$ = 20.0
$R^2_{y\sim x}$ = 0.141
$RMSE_{y\sim x}$ = 22.2
$R^2_{y\sim x}$ = 0.832
$RMSE_{y\sim x}$ = 14.8
$R^2_{y\sim x}$ = 0.397
$RMSE_{y\sim x}$ = 19.0
$R^2_{y\sim x}$ = 0.882
$RMSE_{y\sim x}$ = 12.4
$R^2_{y\sim x}$ = 0.454
$RMSE_{y\sim x}$ = 18.1
Closest relation to the training set
Species
Genus
Family
Order
Class
Phylum
Actual completeness
Actual purity

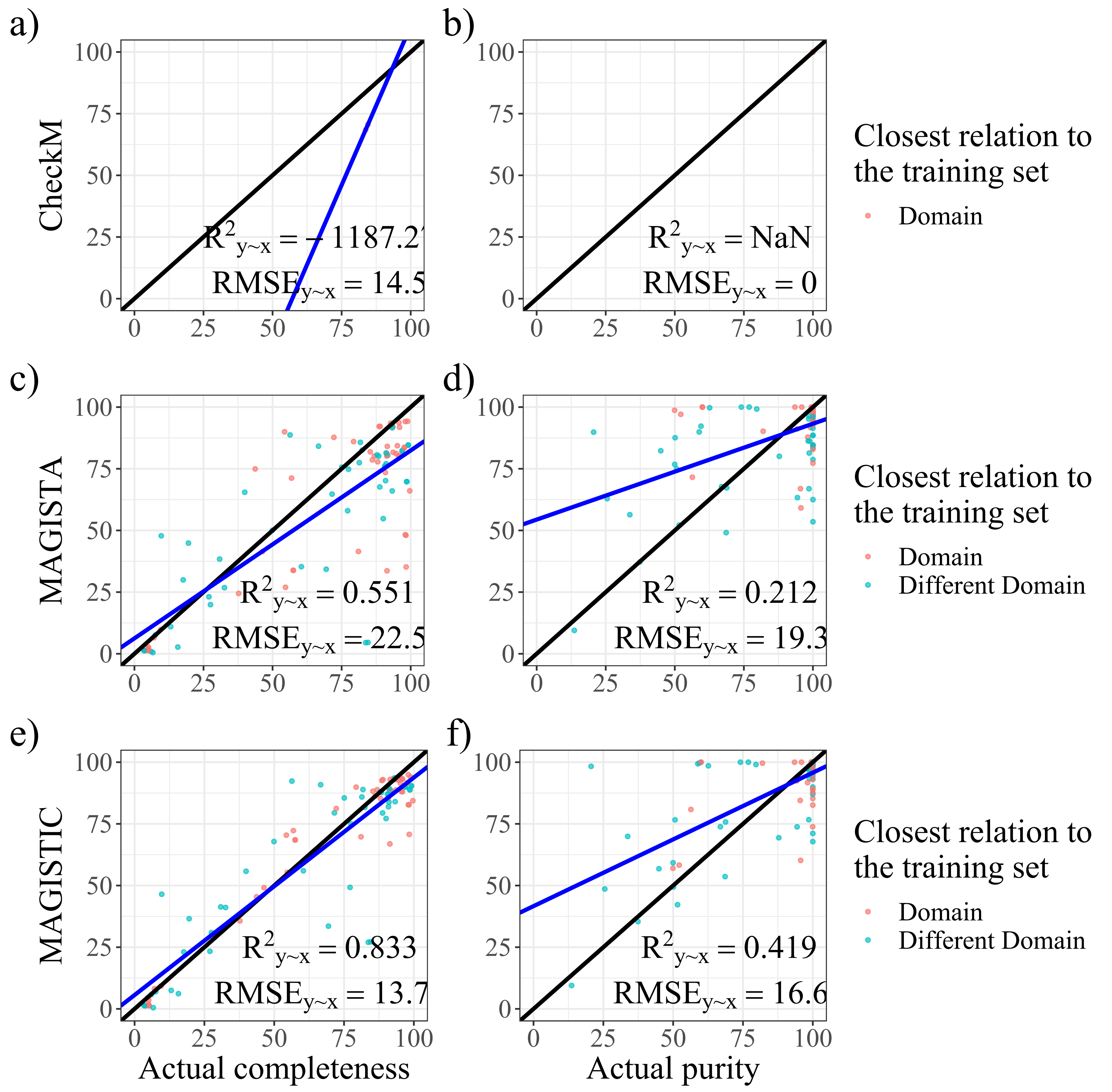
a)
b)
c)
d)
e)
f)
CheckM
MAGISTA
MAGISTIC
$R^2_{y\sim x}$ = − 1187.2
$RMSE_{y\sim x}$ = 14.5
$R^2_{y\sim x}$ = NaN
$RMSE_{y\sim x}$ = 0
$R^2_{y\sim x}$ = 0.551
$RMSE_{y\sim x}$ = 22.5
$R^2_{y\sim x}$ = 0.212
$RMSE_{y\sim x}$ = 19.3
$R^2_{y\sim x}$ = 0.833
$RMSE_{y\sim x}$ = 13.7
$R^2_{y\sim x}$ = 0.419
$RMSE_{y\sim x}$ = 16.6
Closest relation to the training set
Domain
Different Domain
Actual completeness
Actual purity

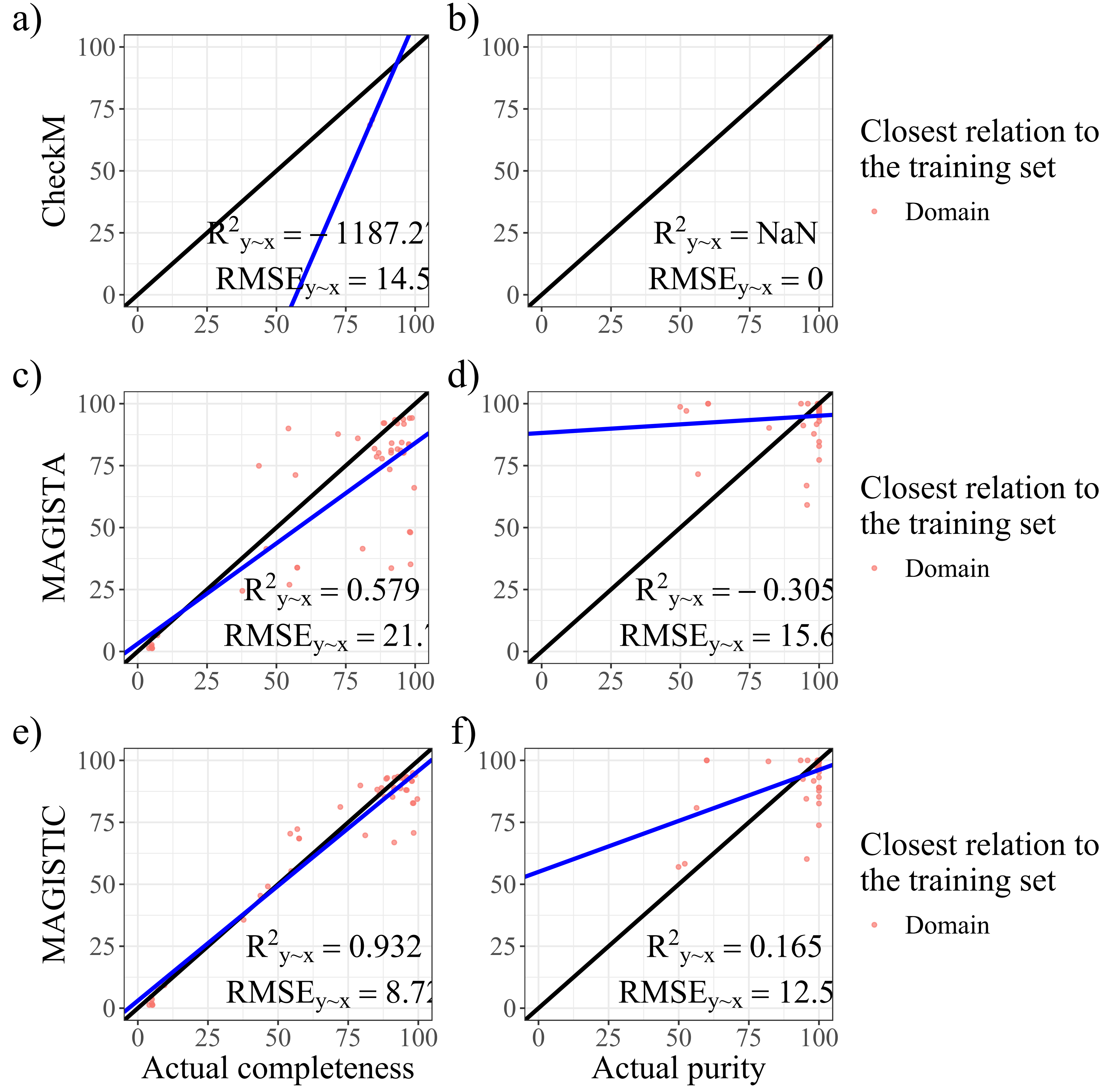

a)
b)
c)
d)
e)
f)
CheckM
MAGISTA
MAGISTIC
$R^2_{y\sim x} = -1187.2$
$RMSE_{y\sim x} = 14.5$
$R^2_{y\sim x} = NaN$
$RMSE_{y\sim x} = 0$
$R^2_{y\sim x} = 0.579$
$RMSE_{y\sim x} = 21.$
$R^2_{y\sim x} = -0.305$
$RMSE_{y\sim x} = 15.6$
$R^2_{y\sim x} = 0.932$
$RMSE_{y\sim x} = 8.7$
$R^2_{y\sim x} = 0.165$
$RMSE_{y\sim x} = 12.5$
Closest relation to the training set
Domain
Actual completeness
Actual purity

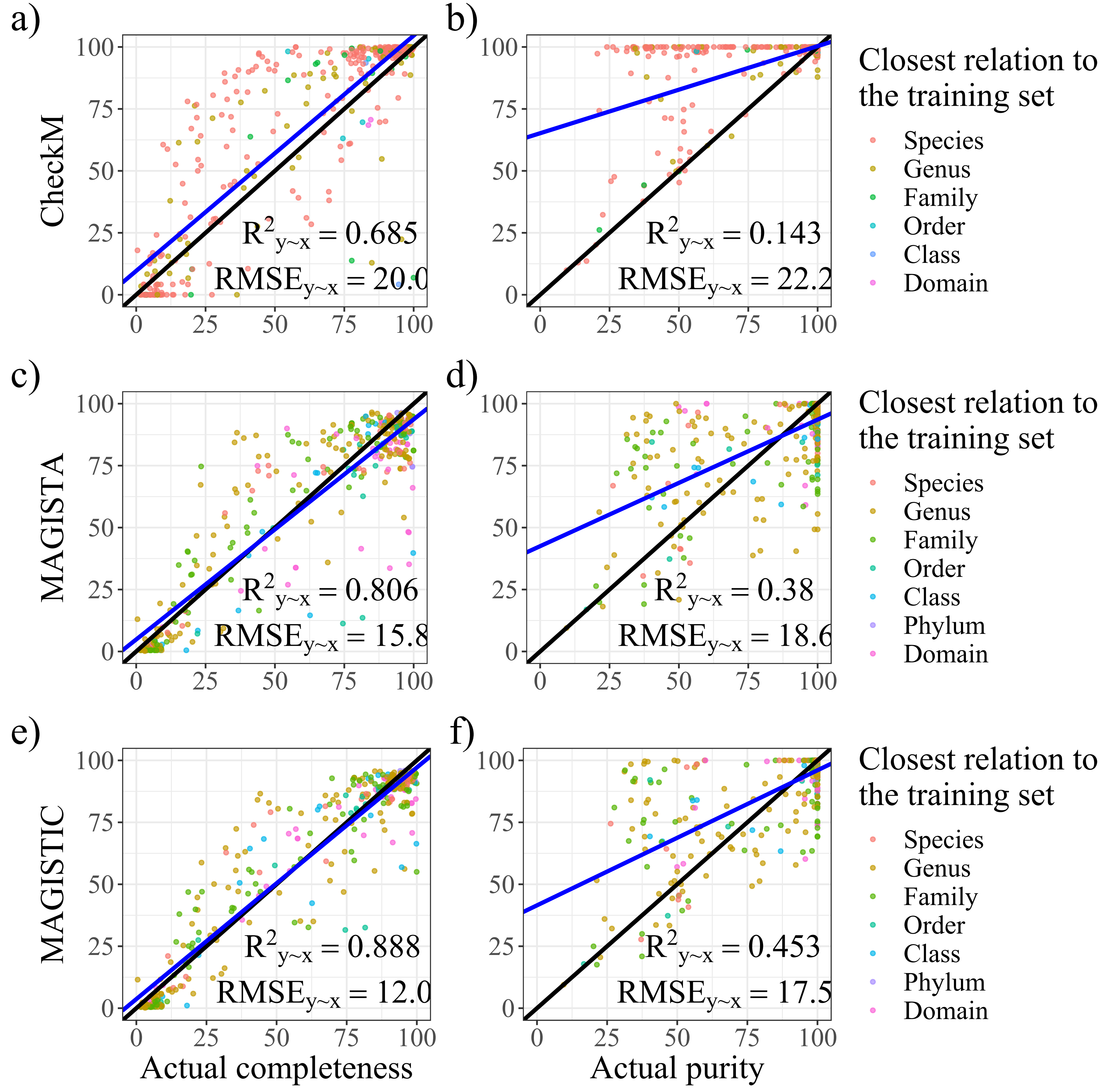

a)
b)
c)
d)
e)
f)
CheckM
MAGISTA
MAGISTIC
$R^2_{y\sim x} = 0.685$
$RMSE_{y\sim x} = 20.0$
$R^2_{y\sim x} = 0.143$
$RMSE_{y\sim x} = 22.2$
$R^2_{y\sim x} = 0.806$
$RMSE_{y\sim x} = 15.8$
$R^2_{y\sim x} = 0.38$
$RMSE_{y\sim x} = 18.6$
$R^2_{y\sim x} = 0.888$
$RMSE_{y\sim x} = 12.0$
$R^2_{y\sim x} = 0.453$
$RMSE_{y\sim x} = 17.5$
Closest relation to the training set
Species
Genus
Family
Order
Class
Phylum
Domain
Actual completeness
Actual purity

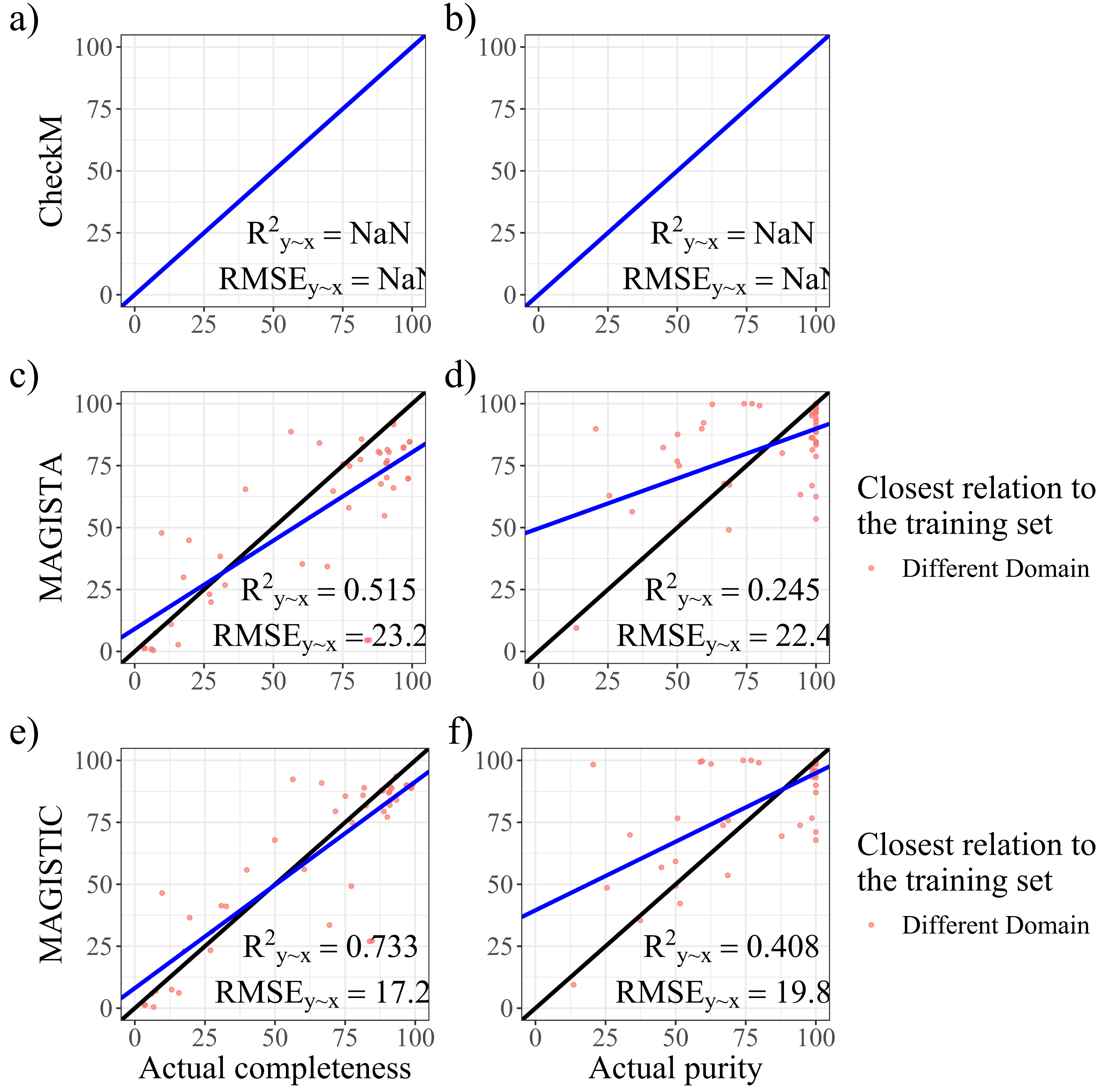

a)
b)
c)
d)
e)
f)
CheckM
MAGISTA
MAGISTIC
$R^2_{y\sim x} = NaN$
$RMSE_{y\sim x} = NaN$
$R^2_{y\sim x} = 0.515$
$RMSE_{y\sim x} = 23.2$
$R^2_{y\sim x} = 0.245$
$RMSE_{y\sim x} = 22.4$
$R^2_{y\sim x} = 0.733$
$RMSE_{y\sim x} = 17.2$
$R^2_{y\sim x} = 0.408$
$RMSE_{y\sim x} = 19.8$
Closest relation to the training set
Different Domain
Actual completeness
Actual purity
